# An extra residue in the electron transfer chain holds the key for the dual functions of an animal-like cryptochrome

**DOI:** 10.1016/j.jbc.2025.110948

**Published:** 2025-11-13

**Authors:** Huaqiang Cheng, Xingbu Ding, Yan-Wen Tan

**Affiliations:** State Key Laboratory of Surface Physics, Shanghai Key Laboratory of Metasurfaces for Light Manipulation, Department of Physics, Fudan University, Shanghai, China

**Keywords:** conformational change, cryptochrome, electron transfer, kinetics, multifunctional protein, photoreduction, redox

## Abstract

Homologous cryptochromes and photolyases have evolved divergently, with cryptochromes acting as photoreceptors to regulate circadian rhythms and photolyases serving as DNA repair enzymes. However, a group of these proteins from algae and fungi exhibit dual functions, including the animal-like cryptochrome from *Chlamydomonas reinhardtii* (*Cra*CRY). In this study, we investigated the mechanism underlying the bifunctionality of *Cra*CRY, using steady-state absorption kinetics, limited proteolysis, and mutagenesis, focusing on the roles of residues in the electron transfer chain beyond the well-known tryptophan triad. Site-directed mutagenesis of Y373, N395, and other residues revealed that charge separation in the electron transfer chain during photoreduction triggers conformational changes. Reducing environments promote the FADH^−^ state required for DNA repair while suppressing the conformational changes needed for circadian regulation. Mutations at Y373 that either enhance or impair photoreduction result in increased dimerization. Our findings suggest that Y373 in the electron transfer chain regulates the dual roles of *Cra*CRY, with the reducing microenvironment determining its functional outcome.

The cryptochrome and photolyase protein family (CPF) is a group of flavoproteins that share homology in sequence and structure but are engaged in diverse functions (1, 2, 3, 4, 5). Generally speaking, most cryptochromes (CRYs) function as photoreceptor proteins to regulate growth and development, entrain circadian rhythms, and even sense geomagnetic fields (5, 6). However, photolyases (PHLs) are known to repair UV-induced DNA lesions. PHLs, which are based on two classes of DNA lesions caused by UV, can be divided into two major groups in which (6–4) PHLs repair 6-4 pyrimidine pyrimidone photoproducts [(6–4) PPs]. Different organisms may have one or more homologs of CRY. For example, *Drosophila melanogaster* has only one kind of CRY, but *Chlamydomonas reinhardtii* has four different CRY homologs. On the phylogenetic tree, one of these four CRYs has been grouped into a cluster of algal and fungal CRYs close to animal CRY2 and insect CRYs; therefore, it is named *C. reinhardtii* animal-like cryptochrome (*Cra*CRY). An updated method of classification of CPF suggests categorizing by functions and lists 10 PHL/CRY groups, with *Cra*CRY classified as a (6–4) photolyase (7). However, a few CRYs from algae and fungi are exceptions because they perform PHL/CRY dual functions, and *Cra*CRY, the focus of this study, is one such example (Fig. 1*A*) (8, 9, 10, 11, 12, 13).

**Figure 1:**
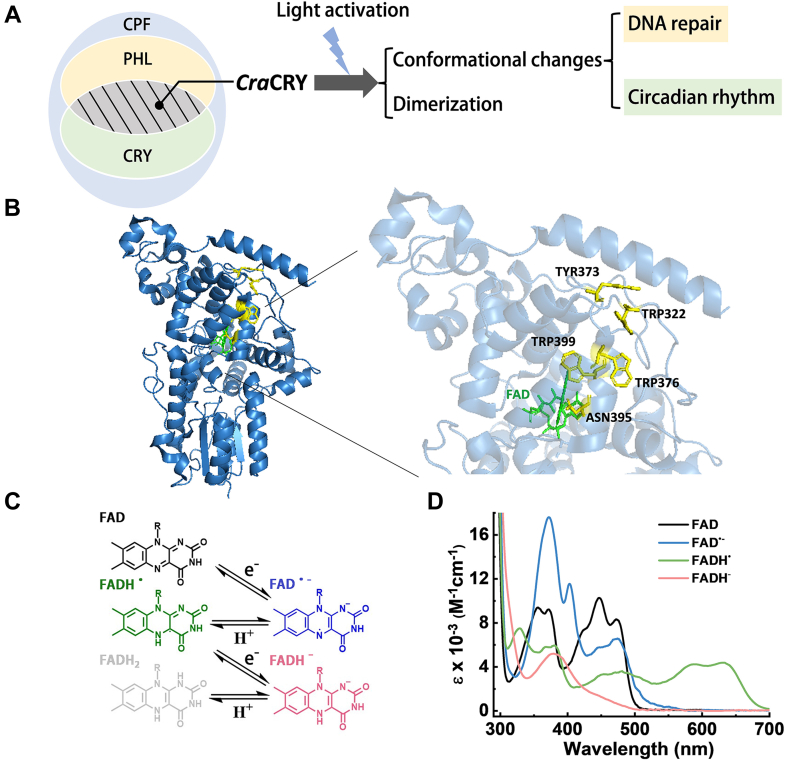
**The different redox states of FAD in *Cra*CRY.***A*, although CRYs belong to the same superfamily as DNA repair photolyases (PHLs), typical CRYs lack the DNA repair activity. *Cra*CRY performs dual functions. At the molecular level, the dimerization, conformational changes and dual functions of *Cra*CRY are dependent on photoreduction. *B*, the overall structure of *Cra*CRY (PDB: 5ZM0). The zoom-in figure on the right depicts the proton donor Asn395, the electron transfer chain consisting of Trp/Tyr, and the location of FAD. *C*, conversion relationships of five redox forms of flavin (FAD). *D*, absorption spectra of the redox forms of FAD in *Cra*CRY. UV–visible spectra were taken from *Cra*CRY-WT, Y373W, or Y373W-N395C.

Cryptochromes are characterized by a photolyase homologous region (PHR) lacking DNA repair activity for most organisms and by a nonconserved C-terminal extension (CTE). Like PHLs, CRYs usually bind a flavin adenine dinucleotide (FAD) cofactor in the PHR domain. The functions of CPFs depend on the reduction and oxidation of FAD induced by light. Among the five potential redox states of flavin, fully reduced hydroquinone (FADH^-^) is the catalytic state for PHLs (13, 14). After association with damaged DNA, PHLs absorb near UV/blue light, and an electron transfers from FADH^-^ to the lesion to break the thymine dimer. In most CRYs, FAD is in the oxidized (FAD_ox_) state when the protein is isolated from cells. *In vitro* photochemistry experiments illustrated that CRYs can be reduced to anionic/neutral radical semiquinone (FAD^•-^, FADH^•^) or FADH^-^ states upon blue light exposure. Meanwhile, the electron is transferred from a chain comprising three or more conserved Trp/Tyr residues, named the Trp triad, during photoreduction. This chain stretches from the FAD to the surface of the protein. In darkness, the reduced CRYs can return to the FAD_ox_ state and the electron acceptor may be oxygen or the Trp/Tyr radical (15, 16, 17, 18, 19).

FAD_ox_ is expected to be the ground state of CRYs because it is the predominant redox form that absorbs blue light. However, experimental evidence suggests that certain proteins can absorb red light *in vivo*, and it is expected that FAD can be chemically reduced to the FADH^•^ state in the subcellular microenvironment (5, 20, 21, 22, 23, 24, 25, 26). The cellular redox potential is approximately −0.15 to −0.42 V, which is maintained predominantly by GSH and NAD(P)H under physiological conditions; thus, it has been argued that the ground states of *Arabidopsis thaliana* CRY1 (*At*CRY1), *Chlamydomonas* photolyase homologue 1, and *Cra*CRY is FADH^•^ *in vivo* (5, 24, 27, 28, 29). However, direct measurements of the electron paramagnetic resonance and absorption of *At*CRY1 overexpressed in Sf21 insect cells revealed that the ground state is FAD_ox_, and the same results were observed for *At*CRY2 and *D. melanogaster* CRY (*Dm*CRY) (30, 31, 32, 33). For *Chlamydomonas*, *Cra*CRY not only repairs the (6–4) PP but also regulates the circadian clock. Our previous work revealed that *Cra*CRY interacts with a circadian protein, Rhythm Of Clock 15 (ROC15), upon blue light excitation (34). Under blue light, *Cra*CRY readily converts to the FADH^-^ state with DTT, as opposed to transitioning to the FADH^•^ state in its absence. The interaction with ROC15 is disrupted in the presence of DTT, as demonstrated by pull-down assays (34). Perhaps DTT acts as a switch between the two functions of *Cra*CRY. The effect of reducing the environment on *Cra*CRY remains to be scrutinized.

Unlike other CRYs with a Trp triad, *Cra*CRY is special in that its electron transfer chain includes a fourth member—tyrosine 373 on the surface of the molecule. *Cra*CRY responds to both blue and red light in *C. reinhardtii,* and transcript accumulation changes accordingly (29). Therefore, FADH^•^ is proposed to be the dark form in *Cra*CRY and is excited to the FADH^-^ form by light. However, the long-lived tyrosyl radical at position 373, believed to be essential for *Cra*CRY functions (35, 36, 37), is formed under both red and blue light. Furthermore, conformational changes, dimerization, and intermolecular interaction can be initiated with UV to blue light (34). In particular, ROC15 interacts with *Cra*CRY in response to blue light. Given that excessive blue light exposure may also facilitate the transition of FADH^•^ to the FADH^-^ state, quantifying the composition of the FAD states during photoreduction is essential.

In general, the signaling functions of CRYs are a direct result of structural changes that are large in scale for the CTE and nonnegligible for the PHR. In both *Dm*CRY and *At*CRY2, light-induced redox changes in FAD modulate CTE conformation and interactions, coupling flavin redox states to conformational changes that regulate downstream signaling functions such as protein binding or oligomerization (3, 38, 39, 40, 41, 42, 43). Unlike *At*CRY2 and *Dm*CRY, the last electron donor of the electron transfer chain in *Cra*CRY is Y373, which dramatically extends the lifetime of the radical state and is postulated to play a central role in the *Cra*CRY light response (19). Conformational changes in the CTE have been directly observed using single molecule Förster resonance energy transfer (34), and structural changes in the PHR have been probed using several analytical techniques (36, 37, 44). It has been proposed that the oxidation of Y373 by proton-coupled electron transfer promotes the light activation of *Cra*CRY (36, 45, 46). However, experimental evidence is lacking.

In this study, we applied steady-state absorption kinetic analysis in combination with a limited proteolysis assay, mass spectroscopy, functional assays, and mutational analysis to probe the mechanism of light-induced photoreduction of *Cra*CRY. Our intention is to scrutinize the linkage between the redox of FAD and the blue-light-induced conformational changes, dimerization, and dual functions of *Cra*CRY. We are especially interested in the roles of the fourth residue on the electron transfer chain, Y373, and the asparagine adjacent to N5 of the isoalloxazine ring, N395. Therefore, mutants of Y373, N395, and a few other residues were investigated under both normal and various reducing environments.

## Results

### Redox kinetics of *Cra*CRY reveal the regulatory roles of Y373 and N395

We constructed Y373 mutants to modulate the photoreduction of *Cra*CRY. Y373 functions as a terminal electron donor during photoreduction (Fig. 1*B*). To assess the role of Y373, we prepared the following mutants: (1) We substituted Tyr with Trp, which is conserved in *Dm*CRY and CRY4 (47). This change is also expected to enhance photoreduction. (2) Tyr was replaced by Arg or Glu. As highly basic and acidic amino acids, these substitutions are expected to disrupt hydrogen bonds. In addition to the terminal electron donor, protonation of FAD should be considered. Therefore, we substituted the residue facing N5 of the isoalloxazine ring, N395, with cysteine and tested the redox kinetics of N395C along with WT and Y373W. From the absorption spectra of WT, Y373W and Y373W-N393C under various conditions, we determined the time evolution characteristics of the four FAD states in *Cra*CRY (Fig. 1, *C* and *D*). Additionally, we referred to the distinct spectra of other CPFs and found that the FADH_2_ form was negligible in the basic buffer used (48). Therefore, the existence of FADH_2_ was not considered in this work.

**Figure 2:**
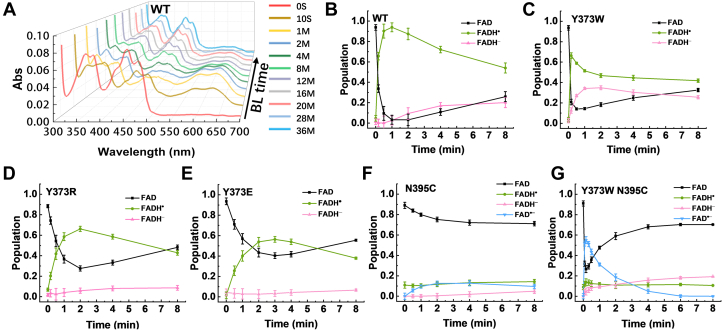
**Photoreduction kinetics of *Cra*CRY-WT and mutants.***A*, time-lapse absorption spectra showing photoreduction of wild-type *Cra*CRY with different exposure times to *blue light* (BL). *B–G*, time-dependent populations of the different FAD states under continuous *blue light* for WT and mutants. The changes in the FAD_ox_ state reached the maximum at 1 min (WT), 1 min (Y373W), 2 min (Y373R), 3 min (Y373E), 4 min (N395C), and 10 s (Y373W N395C). The FAD^•-^ state is negligible for WT and Y373W/E/R. Error bars represent the S.D. from more than three experiments.

The redox kinetics of *Cra*CRY were examined using time-lapse absorption spectra, where absorption at each time point was decomposed into the four flavin redox states (Figs. S1 and S2). First, we measured photoreduction as a function of time by the steady-state absorption spectroscopy of WT and mutant *Cra*CRYs. Notably, the blue light intensity applied here was 100 W/m^2^, which is higher than that used in most other reports, to ensure saturation of exposure. When WT *Cra*CRY was subjected to continuous blue light illumination, the co-factor FADs were readily reduced to FADH^•^ (94 ± 4%), and a small portion was further reduced to FADH^-^ (Fig. 2, *A* and *B*). A key feature of the redox kinetics was that the majority of flavin started to recover to oxidized FAD in approximately 2 minutes even under continuous illumination (Fig. 2*B*). This was illustrated by the absorption increasing in the range between 340 and 500 nm and decreasing in the range between 500 and 700 nm. The same time-lapse absorption spectra were obtained from Y373R, Y373E, and Y373W variants. The results are plotted in Figs. 2, *C*–*E* and S1. Seemingly, the substitutions of charged residues, Y373R and Y373E, did not abolish the photoreduction completely, but the yields of photoreduction (66 ± 3%, 57 ± 3%) were lower than those of the WT based on comparison of the absorption peak ratios at 447 and 587 nm (Figs. 2, *D* and *E* and S1). In contrast, for Y373W, the two absorption peaks at 447 and 587 nm decreased simultaneously from 10 s to 2 min during photoreduction, suggesting the generation of a portion of FADH^-^ (Figs. 2*C* and S1) that accepts two electrons from the Trp chain. *Cra*CRY Y373W was more susceptible to photoreduction than the original Y373. This finding was further supported by the data for the mutants N395C and Y373W-N395C (Fig. 2, *F* and *G*). The N395C mutant showed minimal photoreduction to the FADH^•^ state and was only slightly reduced to the FAD^•-^ state (13 ± 3%), with a modest increase at 371 nm (Fig. S1). However, the absorption change was greater for Y373W-N395C, with a larger portion reduced to FAD^•-^ (55 ± 4%) in this double mutant (Fig. 2*G*). In the two mutants, as with *Dm*CRY, Cys blocked the protonation of FAD (49).

Next, we measured the photoreduction of *Cra*CRYs with reducing agents. GSH and NAD(P)H are the main endogenous reducing agents that balance the redox potential in cells (28). It has been reported that NADH arrests photoreduction of class ⅡDNA photolyases in the FADH^•^ state and that the photoreduction and activity of *At*CRY2 are modulated by small-molecule factors, including NADH (31, 50). In our case, we used three reagent conditions, 10 mM DTT, 10 mM GSH, or 0.1 mM NADH, in the buffer to mimic the surrounding reduction potential of *Cra*CRY in live cells. The time-lapse absorption spectra revealed an enhanced photoreduction efficiency of *Cra*CRY in reducing environments (Figs. S3 and S4).

**Figure 3:**
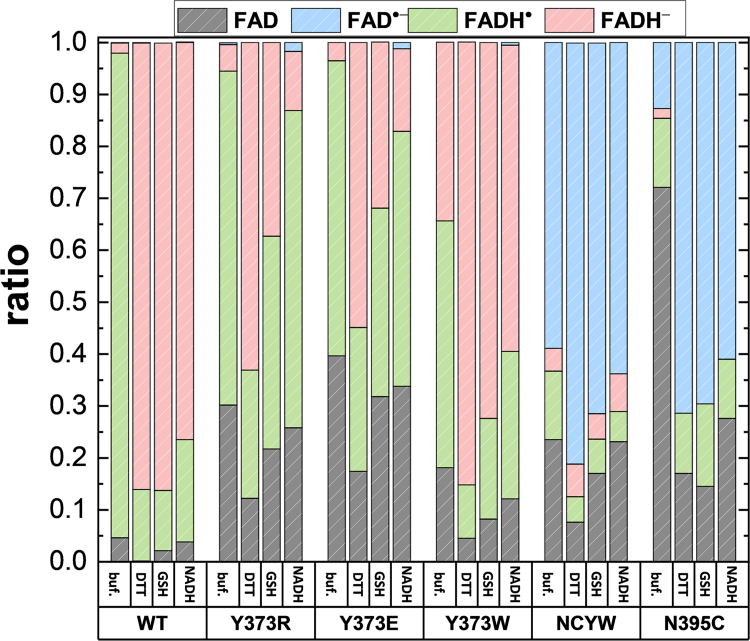
**Photoreduction extents of *Cra*CRY-WT and mutants in the absence (buffer only) or presence of reductants.** The reductants used were 10 mM DTT, 10 mM GSH, and 0.1 mM NADH. The ratios of the four FAD states are derived from photoreduction kinetics measured at the time of maximum change in the FAD_ox_ state. NCYW stands for Y373W-N395C. Error bars and statistical tests are provided in the Supporting Information.

To further elucidate the role of reducing agents, the photoreduction and reoxidation rates in the absence and presence of reducing agents were acquired by fitting the kinetics curves with an exponential decay form, $A\cdot e^{-t/\tau }+B$. Notably, the reduction rate was the reciprocal of the photoreduction lifetime τ, with A and B being the fitting parameters. Additionally, we compared the extent of photoreduction to determine the maximum change in the FAD_ox_ state under illumination. Both the photoreduction rate (Fig. S8*A* and Table S1) and extent (Fig. 3, Tables S2 and S3) are the quantitative descriptions of the photoreduction efficiency. In the absence of reducing agents, only a small amount of *Cra*CRY-WT was reduced to the FADH^-^ state and ∼94% FADH^•^ was generated. By contrast, if *Cra*CRY-WT was illuminated with DTT or GSH, ∼85% of the product was in the FADH^-^ state. The results are summarized in Figure 3. For *Cra*CRY-WT, GSH and NADH played similar roles to DTT in enhancing the accumulation of FADH^-^ during photoreduction. The same experiments were performed on mutants treated with reducing agents as shown in Figure 3. In general, the reducing capacity of low-concentration NADH was weaker than that of GSH or DTT, with DTT being the strongest among the three. With reducing agents, the Y373 mutants were fully reduced to the FADH^-^ state, whereas any mutant with N395C was stuck at FAD^•-^. Owing to the Tyr at position 373, the state difference observed in *Cra*CRY-WT caused by DTT was the greatest. This promotion of reduction could be observed in WT and N395C. Compared with Y373R (0.54 ± 0.09 min) and Y373E (1.10 ± 0.26 min), *Cra*CRY-WT showed a faster photoreduction rate (0.22 ± 0.06 min), whereas the WT photoreduction rate was slower than that of Y373W (0.06 ± 0.01 min) (Fig. S8). And the increase in photoreduction rate caused by DTT was not significant. *Cra*CRY-WT had the longest reoxidation lifetime (21.4 ± 2.9 min) after blue light illumination (Figs. S5–S8). The reoxidation rates of N395C and Y373W-N395C (1.2 ± 0.1 min) were significantly faster, which was attributed to the stabilization of reduction through protonation. Furthermore, the reoxidation lifetimes of Y737W and Y373R/E were extended by DTT, as DTT quenched the radical. Overall, the reduction potential of the environment could prevent reoxidation and enhance photoreduction, with Y373 amplifying these effects.

### Conformational changes in *Cra*CRY are initiated by light induced charge separation in the photoreduction of FAD

**Figure 4:**
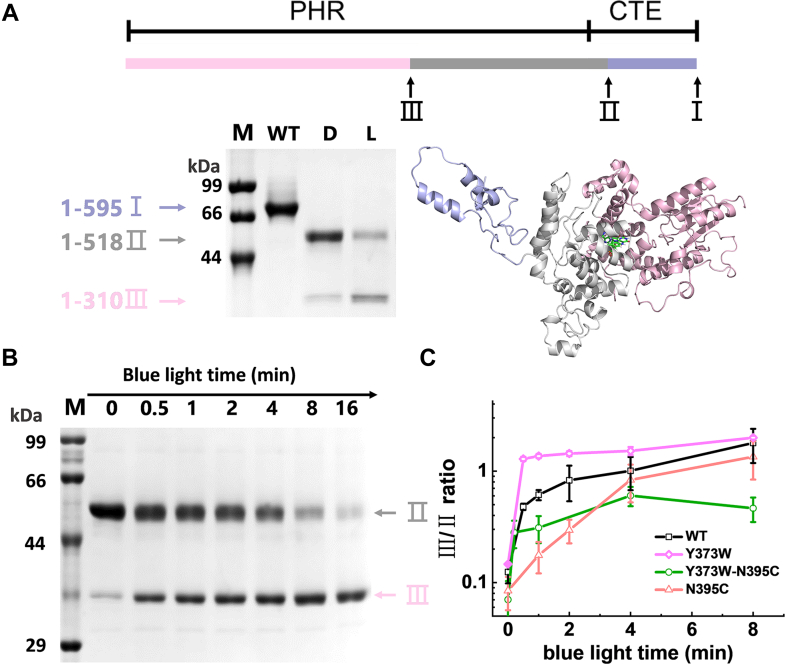
**Limited proteolysis of *Cra*CRY and kinetics of conformational changes for wild-type *Cra*CRY and mutants in the absence of reductants.***A*, MS analysis reveals proteolysis sites of *Cra*CRY-WT in the *dark* and under *blue light*, as shown by the schematic and cartoon. *B*, *Cra*CRY-WT was exposed to *blue light* for different times before trypsin was added and incubated in the *dark* for another 30 min. Changes in proteolysis are shown by SDS-PAGE. *C*, kinetic analysis of the conformational changes in the WT and mutants upon blue light illumination, as indicated by the band III/II ratio in proteolysis in the absence of reductants. Error bars reflect the S.D. for n ≥ 3. M, D, and L represent marker, dark, and lit, respectively.

We used proteolysis to investigate conformational changes in *Cra*CRY. Limited proteolysis associated with mass spectrometry is a simple yet efficient method to detect protein conformational changes (17, 39, 47). Purified *Cra*CRY-WT, kept either in the dark or under blue light, was subjected to limited proteolysis with trypsin. The products were subsequently analyzed by SDS-PAGE. Two main bands were observed in all proteolytic samples tested (Figs. S9–S12). The bands were analyzed by mass spectroscopy, and the predicted digestion sites were mapped and plotted in Figure 4*A*. Band II was cleaved at R518, and it contains 28 additional residues from the CTE, suggesting that this region is more structured in full-length *Cra*CRY. The lower band III (see Fig. 4, *A* and *B*) became darker after *Cra*CRY-WT was illuminated for an extended period, which is an indicator of conformational change. Most of the CTE of *Cra*CRY was digested completely by trypsin, making it barely detectable on the gel. The light-sensitive cleavage site identified by bands III and II was R310, which is the initial residue of the R310-N325 loop near Y373 and W322. The photoinduced conformational change of this loop agreed with the hydrogen/deuterium exchange mass spectrometry (HDX-MS) data reported by Sophie *et al.* (36).

We applied the proteolytic assay to examine the kinetics of light-induced conformational changes in relation to the FAD redox state. Because the intensity of band III increased with increasing blue light illumination time, the ratio of bands III to II was used to indicate the kinetics of the *Cra*CRY conformational changes. Although *Cra*CRY-WT reached the saturated FADH^•^ state in 2 minutes, the conformational change ratio increased over 16 min with continuous blue light exposure (Fig. 4, *B* and *C*). The conformational changes of the other mutants, N395C, Y373W, and Y373W-N395C, were comparable at the same cleavage sites (Fig. S10), with Y373W and Y373W-N395C exhibiting slightly more rapid conformational changes (Fig. 4*C*). Much to our surprise, the conformational change was initiated by photoreduction, yet the connection between the FAD redox state and the *Cra*CRY conformational changes was weak. As shown in Figure 4*C*, although the ratios of bands III/II for different mutants increased at different rates, most of them eventually reached similar levels (except for Y373W-N395C). From the previous experiments, we know that the photoreduced states of N395C and Y373W-N395C were FAD^•-^ without protonation, but they exhibited comparable levels of conformational changes. Flavin states are decoupled from conformational changes in *Cra*CRY.

During photoreduction, the electron transfer chain is the electron donor, whereas FAD is the acceptor. What if the electron transfer chain is not the final electron source? Reducing agents in the buffer, such as GSH or DTT, can act as external electron donors to quench the Tyr or Trp radicals (25, 26, 51, 52). A proteolysis assay of *Cra*CRY under different reducing agents was performed to address this question.

**Figure 5:**
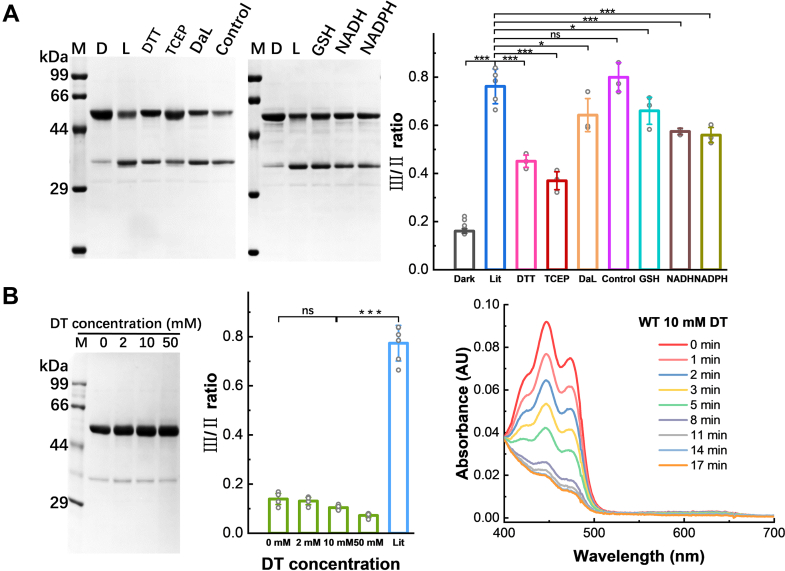
**Limited proteolysis and conformational changes of *Cra*CRY in the presence of reductants.***A*, limited proteolysis of *Cra*CRY-WT in different reducing environments upon *blue light* illumination. M: marker; D: dark; L: lit; DTT: the sample was exposed to *blue light* for 3 min in 50 mM DTT and then diluted 20-fold before incubation with trypsin; DaL:50 mM DTT was added after *blue light* illumination and incubated for 10 min before proteolysis; Control: the sample exposed to *blue light* was incubated with trypsin in buffer containing 2.5 mM DTT. *B*, *Cra*CRY was chemically reduced by DT in the *dark*, but no conformational change was observed. The samples were incubated with DT for 12 min and then diluted 10-fold before incubation with trypsin. Data are averaged from at least three independent experiments, and error bars indicate S.D. The data were analyzed by one-way ANOVA with Dunnett’s *post hoc* test; ∗*p* < 0.05, ∗∗∗*p* < 0.001; ns, not significant.

As shown in Figure 5*A*, the III/II ratio of *Cra*CRY in the reducing buffer was lower than that in the normal buffer. The conformational change ratios decreased as the reducing capacity increased. After illumination, the band III/II ratio of *Cra*CRY in TCEP, the strongest reducing agent tested here, was the lowest. DTT had an effect similar to that of TCEP in inhibiting conformational changes. Dithionite (DT), at concentrations above 10 mM, could reduce *Cra*CRY even without light excitation. Unexpectedly, *Cra*CRY treated with DT hardly underwent any conformational change (Figs. 5B and S13). When the Tyr radical was reduced in the presence of reductants, conformational changes were inhibited, and the extent of inhibition correlated with the reducing strength (Fig. 5).

To further evaluate the relationship between the electron transfer chain and conformational changes, we generated several mutations in the vicinity of Y373. We identified five mutants in total. Among them, the three Y373 mutants had Y373 substituted with the nonpolar amino acid Ala, positively charged Arg, and negatively charged Glu. Unlike Tyr and Trp, the three mutants, which lack an aromatic ring in their side chains, exhibited high hydrolysis efficiency even without light illumination (Fig. S12). In addition to residue 373, E377 and E381 are close to R310, the initial residue of the loop near Y373 and the cleavage site of band III. However, the positively charged mutants E377K and E381K had opposite effects. E377K exhibited greater hydrolysis efficiency, whereas E381K tended to resist hydrolysis in the dark. The direct effect of the surface E377K mutation was hydrogen bond rearrangement around R310 (Fig. S12) (12). Thus, the R310-N325 loop mediated the oxidation of Y373 and the *Cra*CRY conformational changes.

### Interaction and DNA repair activity are regulated by Y373 and reducing environments

**Figure 6:**
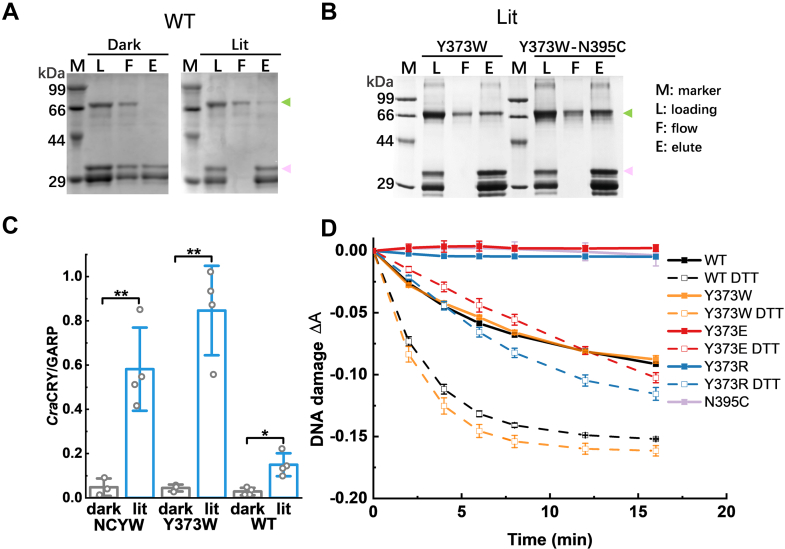
**Interactions of *Cra*CRY mutants with ROC15 and photolyase activity.***A–C*, pull-down assay using SDS-PAGE of *Cra*CRY WT and mutants under *blue light* in the absence of reducing agents. The *green arrows* mark the band of *Cra*CRY. The *pink arrows* mark the band of GST tagged ROC15(GARP). NCYW stands for Y373W-N395C. The interaction was quantitatively assessed based on the ratios of *Cra*CRY to GST–GARP band intensities. Data are shown as mean ± S.D. from at least three independent experiments. ∗*p* < 0.05, ∗∗*p* < 0.01; two-tailed, unpaired *t* test. *D*, photolyase activities of *Cra*CRY-WT and mutants in the presence or absence of DTT as a reducing environment. The decreasing absorbance at 325 nm indicates the repair of (6–4) PP. Error bars reflect the S.D. for n ≥ 3.

*Cra*CRYs not only interact with ROC15 to regulate the circadian clock but also repair DNA lesions in *Chlamydomonas*. Both functions are dependent on blue light (12, 29). We inspected the functions of the above mutants to uncover the underlying mechanism. GST pull-down methods were used to identify interactions of mutants with ROC15. In our previous study, we reported that reductants like DTT and TCEP disrupt the interaction with GARP (the active domain of ROC15) with or without blue light (34). In contrast, the mutants Y373W, Y373W-N395C, Y373R, and Y373E could interact with GARP in the absence of reductants (Figs. 6, *A*–*C* and S14). The results showed that the specific FAD state is not a determining factor for the interaction.

To compare the DNA repair activities of wild-type *Cra*CRY and mutants, we performed *in vitro* assays using an established method (12, 53). The assays used spectrophotometry, with a decrease in absorption at 325 nm indicating the repair of (6–4) PP during the incubation of *Cra*CRY with a (6–4) PP comprising oligo(dT)_18_ under illumination at 450 nm. The activities of Y373R and Y373E are lower than those of wild-type *Cra*CRY and Y373W, which are correlated with their photoreduction efficiencies (Fig. 6*D*). The repair rates of wild-type *Cra*CRY and Y373W with DTT were significantly higher than those without DTT, aligning with the proportion of the FADH^-^ state. *Cra*CRY exhibited higher activity in repairing DNA damage in the presence of an external electron donor.

## Homodimerization is regulated by Y373 and DTT

Photo-oligomerization is an evolutionarily conserved photoreaction characteristic of CRY photoreceptors (42). To elucidate the relationship between photoreduction and dimerization, we utilized size exclusion chromatography (SEC) to separate oligomers, dimers, and monomers. The integrated area of the elution peaks was used to define the oligomeric fraction within the entire *Cra*CRY elution area. Within a specific concentration range, the dimerization of *Cra*CRY increased to approximately 20%, representing the maximum dimerization ratio of *Cra*CRY-WT at a concentration of 16 mg/ml (Fig. S15). We assessed the dimerization of *Cra*CRYs at equivalent concentrations and evaluated the time-lapse changes induced by blue light illumination. Notably, the dimerization of wild-type *Cra*CRY was weaker than that of the mutants, with the Y373R and Y373E variants showing an accumulation of dimers of up to approximately 50% (Figs. S16 and S17). Mutation at Y373 altered the ability of *Cra*CRY to form dimers. With increasing illumination, the dimerization of all *Cra*CRY mutants increased in the first 4 min and stagnated later. All four Y373 mutants presented a relatively higher percentage of dimers, with the proportion of the dimer peak area exceeding 30%. In contrast, the dimer percentage of N395C matched that of wild-type *Cra*CRY. In the presence of DTT, photoinduced dimerization was prevented (Fig. S15). Alterations at Y373 and reducing environments could also modulate the dimerization of *Cra*CRY.

To focus on the characteristics of the dimer, we collected samples of the dimer peak of *Cra*CRY using SEC and kept them in the dark. We found that the dimers of wild type still eluted as dimers in a second round of SEC (Fig. S18, *A*–*C*). Once the dimer formed, it was stable and minimally dissociated back to monomers after reoxidation. We subjected the dimer and monomer samples to a second round of blue-light photoreduction. After several hours in the dark, the FAD states of both monomeric and dimeric *Cra*CRY predominantly reverted to their oxidized forms. The responses of the dimer and monomer to subsequent blue light illumination differed significantly. While most monomeric *Cra*CRY could still respond to blue light and undergo reduction, few dimers could respond (Fig. S18, *D*–*F*). The stabilized dimer prevented *Cra*CRY from responding to blue light.

## Discussion

*Cra*CRY is a special cryptochrome since it is bifunctional with both photolyase and circadian entrainment activities. Both functions depend on light excitation. At the molecular level, blue light can induce photoreduction, conformational changes, dimerization, and interactions with ROC15 of *Cra*CRY. Using site-directed mutagenesis combined with photochemistry, proteolysis, and SEC, we studied the mechanistic effects of *Cra*CRY photoreduction and its potential causal relationship with functions. All experiments were conducted under steady-state conditions. Steady-state measurements reflect the equilibrium result after forward and reverse reactions cancel each other out, whereas transient measurements focus on unidirectional reactions. They are complementary for studying kinetic processes. In our redox study, both forward and backward electron transfers occur simultaneously under blue light, although forward transfer predominates. Blue light exposure shifts the equilibrium toward the forward direction, allowing us to monitor the accumulation of specific molecular states. Steady-state measurements have been widely applied in studies of various cryptochromes and photolyases (17, 47, 49, 54, 55). Although *in vitro* systems cannot fully replicate the cellular environment, they allow comparison of wild-type and mutant *Cra*CRY under controlled conditions. These setups effectively reveal redox behavior and conformational changes. A previous study on *At*CRY2 confirmed that *in vivo* modulation by cellular compounds can be mimicked with *in vitro* measurements (31).

**Figure 7:**
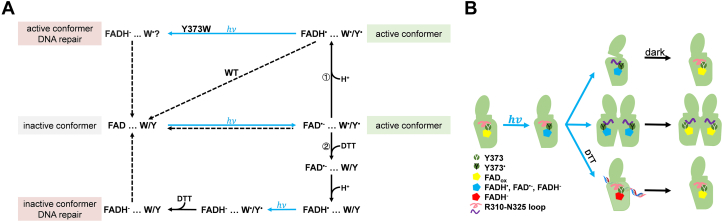
**Regulatory mechanism of *Cra*CRY in switching between dual functions.***A*, a proposed model for the photoreaction of *Cra*CRY in the absence or presence of DTT. Y/W indicates Tyr or Trp at position 373. The final states for WT and N395C are [FADH^•^ Tyr^•^] and [FAD^•-^ Tyr^•^] in the absence of DTT, respectively. The final state for WT is [FADH^-^ Tyr] in the presence of DTT. The final state for Y373W may be mixture of [FADH^-^ Trp^•^] and [FADH^•^ Trp^•^] in the absence of DTT. The final state for Y373W is [FADH^-^ Trp] in the presence of DTT. The final state for Y373W-N395C is [FAD^•-^ Trp^•^] in the absence of DTT. The reduction of Tyr (path 2) is rapider than the protonation of FAD^•-^ (path 1) in the presence of DTT. *B*, upon *blue light* illumination, electron transfer occurs from Y373 to FAD. In the presence of DTT as an external electron donor, the Y373^•^ radical is quenched, and *Cra*CRY converts to the FADH^-^ state to repair DNA. In the absence of DTT, the oxidation of Y373 directly triggers conformational changes, dimerization, and interactions. The reduced *Cra*CRY reverts to its oxidized state in the *dark*.

Y373 is believed to be essential for *Cra*CRY. We constructed the mutants Y373W, Y373R, Y373E, N395C, and Y373W-N395C for *Cra*CRY based on the findings of previous studies on *Cra*CRY and other species, such as *Dm*CRY, *At*CRY1/2, and *Cl*CRY4. In the dark, the purified mutants were in the FAD_ox_ state, the same as the wild type. Upon exposure to blue light, they convert to FAD^•-^, FADH^•^, or FADH^-^ under steady-state conditions (48). Y373W and WT exhibited faster and more thorough photoreduction than Y373R/E, indicating that the Tyr/Trp tetrad is more effective than the triad. This is consistent with the results of Nohr (19). Once returned to darkness, these mutants gradually revert to the oxidized state. The mutants and wild-type *Cra*CRY exhibited varying photoreduction rates and extents, as well as different reoxidation rates, under different reducing reagents. Photoreduction in *Cra*CRY involves two half-reactions: reduction of FAD and oxidation of Trp/Tyr. Based on previous transient absorption and transient electron paramagnetic resonance experiments on cryptochromes and photolyases (25, 26, 45, 51, 52), the formation of radical pairs in *Cra*CRY can be inferred under specific conditions (Fig. 7*A*). These mutants help to reveal the relationship between FAD photoreduction, oxidation of Tyr, and the functions of *Cra*CRY.

It is generally believed that the conformational changes in *Cra*CRY, as in other cryptochromes, are dependent on the redox conditions of FAD. In *Cra*CRY, four residues, Trp399, Trp376, Trp322, and Tyr373, constitute the electron transfer chain. Upon blue light illumination, most *Cra*CRY-WT is reduced to the FADH^•^ state in nonreducing buffer or FADH^-^ in reducing buffer. Tyr or Trp at position 373 increases the sensitivity of *Cra*CRY to the redox potential of the environment (Fig. 3). Furthermore, conformational changes determined by proteolysis are suppressed in reducing buffer. There are two effects of reducing agents as external electron donors on *Cra*CRY-WT: reducing FAD_ox_ to the FADH^-^ form with light and quenching the electron donor Y373 (Fig. 7*A*). Do the conformational changes directly result from different FAD states? In our work, N395C and Y373W-N395C could only be converted to the FAD^•-^ state due to the loss of protonation. To our surprise, the conformational changes of these mutants persist, although mostly at a slower rate. In contrast, DT can reduce *Cra*CRY to FADH^-^ in the absence of light but cannot induce conformational changes. Combining these findings, we conclude that the charge of flavin is not a key feature for conformational changes in *Cra*CRY, unlike *Dm*CRY (17, 41). These results suggest that FAD states are decoupled from conformational changes in *Cra*CRY during photoreduction, although further investigation is needed for more direct evidence.

Nohr *et al.* reported that *Cra*CRY mutants W322F and Y373F-W376F exhibit no photoreduction activity in the presence of 10 mM DTT (19). This implies that FAD located at the center of *Cra*CRY cannot accept electrons from solution and that the Trp-Tyr chain is the only direct electron donor (Fig. S13, *E* and *F*). We deduced that both electrons of FADH^-^ in *Cra*CRY-WT come from DTT through the electron transfer chain, because the Tyr radical is quenched (Fig. 7*A*). Unlike the WT, a larger portion of Y373W converts to the FADH^-^ state in the absence of reducing agents, indicating that this substitution enables the electron transfer chain to donate one more electron than the WT (Fig. 7*A*). Thus, the photoreduction rate and conformational change rate of Y373W would be higher (Fig. 4*C*). The same effect was observed for N395C and Y373W-N395C. Furthermore, reducing agents inhibit conformational changes by quenching the electron donor. These results indicate that the oxidation of Tyr or Trp is involved in triggering the conformational changes.

*Cra*CRY conformational changes, monitored by limited proteolysis, manifest as a different ratio of two bands, and light exposure makes *Cra*CRY easier to digest by trypsin. Peptide fragments determined by mass spectra indicate that the CTE fragment after R518 is readily digested, which suggests a rigid PHR domain and a relatively flexible CTE. In addition, the major light-activated cleavage sites are R310 and R518. Mapping fragments to the 3D structure indicates that the R310-R518 region includes the first 28 amino acids of the CTE, the alpha helix α22 connecting PHR to CTE, and the R310-N325 loop in the PHR. These are all in the vicinity of site Y373. These results are in accord with those obtained from small angle X-ray scattering (SAXS)-driven simulations and HDX-MS measurements on *Cra*CRY (34, 36). If Tyr is substituted with Arg or Glu, mutants lacking aromatic rings are efficiently digested in the dark, which implies an altered structure of R310-R518. These findings demonstrate that the rearrangement of the R310-R518 structure results from the oxidation of Y373 induced by blue light illumination. However, the detailed dynamics of the conformational changes in full-length *Cra*CRY remain to be determined.

Another effect, which may be the consequence of conformational change, is the circadian entrainment function of *Cra*CRY. In our previous study, upon blue-light illumination, a fraction of *Cra*CRY was shown to interact with the circadian-related transcription factor ROC15, and reducing agents abolish the interactions (34). Here, we performed pull-down assays on the electron transfer-strengthening mutant, Y373W, and the electron transfer-compromising mutants, Y373R, Y373E, and Y373W-N395C. The results show that the charged mutants Y373R and Y373E, which compromise the electron transfer chains, maintain interaction with ROC15, whereas mutants containing Y373W increase the interaction strength compared with WT. In addition, the conformations of *Cra*CRY under blue light are different from those with reducing agents. This may imply that the conformational changes are necessary for interaction. On the other hand, in the DNA repair assays, only the Y373W mutant displays a similar function to that of the WT. The charged mutants, Y373E and Y373R, presented diminished repair function and were restored only to the WT level by the addition of the reducing agent DTT. In contrast, N395C has completely impaired DNA repair ability because it rarely reaches the DNA repairing FADH^-^ state (13). Summing up both experiments, Y373 plays a critical role in balancing the capabilities of this dual-function protein. A few other species in this (6–4) PHL category have also been determined to be bifunctional (7, 11, 56). Based on multiple sequence alignment, Trp is still the most common residue at positions corresponding to Y373. Other commonly observed amino acids are Tyr and Phe. It would be interesting to examine whether those species with Phe at this residue retain bifunctional capability.

Moonlighting proteins are found to have two or more different functions, and the switch may involve the cellular location, cell type, oligomeric state, or the cellular concentration of a ligand, substrate, cofactor or product (57, 58, 59). Most moonlighting proteins are conserved enzymes that have acquired new nonenzymatic functions through evolution (60). *Cra*CRY is likely at the stage of evolution where it has developed circadian regulation activity while retaining its photolyase activity. Some proteins can serve multiple functions depending on the microenvironment, including defending against environmental stress (58, 61, 62). In the case of *Cra*CRY, a reducing condition in the microenvironment is the potential trigger at Y373 in the electron transfer chain to switch from regulating circadian rhythm to the repair of DNA (Fig. 7*B*).

Moreover, mutations at Y373 can alter the extent of dimerization of *Cra*CRY, providing an example for further exploration of conserved oligomerization in CRYs. In irreversibly dimerized *Cra*CRY, FAD can revert to the FAD_ox_ state but cannot be photoreduced during a second round of illumination (Fig. S18). This may be one reason for the return of FAD to the oxidized state observed in Figure 2.

The true cellular ground state of flavoproteins is still under debate, although purified CRYs are usually in the FAD_ox_ state in the dark. Because the midpoint redox potentials of FAD_ox_/FADH^•^ and FAD_ox_/FAD^•−^ have been estimated to be comparable to or even higher than the redox potential of the cell cytoplasm, the ground states of *At*CRY1 and *Dm*CRY were argued to be FADH^•^ and FAD^•−^, respectively (5, 21, 23, 63, 64). Two different values for FAD/FAD^•−^ in *Dm*CRY have been reported (−316 mV and 125 mV), and the midpoint potential of FAD/FADH^•^ in *At*CRY1 is approximately −143 mV to −153 mV (23, 63, 64). Another piece of supporting evidence is that *At*CRY1 responds to green light in living cells, and the same situation occurs with *Cra*CRY in response to red light in *C. reinhardtii* cells (23, 29). However, GSH, NAD(P)H, and DTT cannot chemically reduce *Cra*CRY *in vitro,* even though they have a redox potential of −0.24 V, −0.32 V, and −0.33 V at pH 7.0, respectively (28). And DT reduces *Cra*CRY to the FADH^-^ state in the dark. There is no stable FADH^•^ state for *Cra*CRY in reducing buffers. Based on these data, the redox potentials of the *Cra*CRY flavin remain undetermined, although the FAD redox potential of *Cra*CRY may be close to that of *Dm*CRY (−316 mV). On the other hand, reducing environments promoted the conversion of *Cra*CRY to the FADH^-^ state, enabling the repair of (6–4) lesions. Meanwhile, conformational changes and interactions with ROC15 were disrupted. Redox potentials vary significantly across subcellular compartments. Taken together, the two different functions of *Cra*CRY may be activated from different “dark states” of flavin, and the cellular microenvironment may invoke either function. The connections between the states and functions of *Cra*CRY in living cells need further scrutiny, and more experimental evidence await.

In conclusion, steady-state absorption kinetic analysis, in combination with a limited proteolysis assay, functional pull-down assays, and (6–4) DNA lesion repair assays were performed on wild-type *Cra*CRY and its mutants to probe the regulatory mechanism in bifunctional *Cra*CRY. One of the major findings is that during photoreduction the *Cra*CRY-WT, in which the FADH^•^ state dominates, and Y373W-N395C, in which the FAD^•-^ state dominates, undergo comparable conformational changes as monitored by the proteolytic sensitivity assay. These results implicate the special role of Y373, the fourth residue on the electron transfer chain, which holds the key for the dual functions of *Cra*CRY. The oxidation of Y373 directly triggers conformational changes, potentially resulting in circadian entrainment. In a reducing environment, however, the oxidation of Y373 is suppressed, which facilitates the reduction of flavin to FADH^-^, making *Cra*CRY ready for DNA repair.

### Experimental procedures

#### Protein expression and purification

The genes encoding wild-type *Cra*CRY and mutants were cloned into the pET-28a vector and expressed in *E. coli* BL21(DE3). All proteins were expressed and purified as previously described (34). The last step of protein purification was run on a Superdex 200 Increase 10/300 GL column under darkness in a buffer containing 50 mM HEPES (pH 7.4) and 150 mM NaCl. Afterward, protein concentrations were measured by absorption at 280 nm.

#### Steady-state UV-vis absorption and photoreduction assays

Absorption spectra were recorded using an Agilent 8453 spectrophotometer. The protein samples were measured in buffer containing 50 mM HEPES (pH 7.4) and 150 mM NaCl. In the presence of reducing agents, 10 mM DTT, 10 mM GSH, 0.1 mM NADH or 0.1 mM NADPH was added to the buffer. The spectra were measured at room temperature after different illumination durations using a high-power LED emitting at 450 nm (100 W/m^2^). And the LED was placed 1 cm above the cuvette to avoid overheating. To obtain the spectrum of the FAD_ox_ state, wild-type *Cra*CRY was measured in buffer containing HEPES (pH 7.4) and 150 mM NaCl in the dark. To obtain the spectrum of FADH^•^ state, wild-type *Cra*CRY was measured in buffer containing HEPES (pH 6.6) and 150 mM NaCl after 1 min of blue light illumination, which reached the maximum absorbance at 587 nm. To obtain the spectrum of the FAD^•-^ state, Y373W-N395C was measured in buffer containing HEPES (pH 7.4), 150 mM NaCl, and 10 mM DTT after 10 s of blue light illumination, which reached the maximum absorbance at 371 nm. To obtain the spectrum of FADH^-^ state, Y373W was measured in buffer containing HEPES (pH 7.4), 150 mM NaCl and 10 mM DTT after 2 min of blue light illumination, and the absorbance minima were seen at 447 nm and 587 nm. When Y373W and Y373W-N395C were measured, a small amount of absorption between 500 nm and 700 nm, associated with the FADH^•^ state, was proportionally converted to the corresponding FADH^-^ and FAD^•-^ states. To determine the molar absorption coefficients, the absorbance values for all four states were calibrated against the FAD_ox_ state and the *Cra*CRY concentration and then compared with spectral profiles of other CPF proteins (48). A given absorption spectrum during photoreduction or reoxidation was fitted using below.

$${ABS}_{\exp}=a_{1}\cdot \varepsilon_{\left[{FAD}_{ox}\right]}+a_{2}\cdot \varepsilon_{\left[{FAD}^{\cdot -}\right]}+a_{3}\cdot \varepsilon_{\left[{FADH}^{\cdot }\right]}+a_{4}\cdot \varepsilon_{\left[{FADH}^{-}\right]}$$

$$\left[{FAD}_{ox}\right]=\frac{a_{1}}{a_{1}+a_{2}+a_{3}+a_{4}}$$

$$\left[{FAD}^{\cdot -}\right]=\frac{a_{2}}{a_{1}+a_{2}+a_{3}+a_{4}}$$

$$\left[{FADH}^{\cdot }\right]=\frac{a_{3}}{a_{1}+a_{2}+a_{3}+a_{4}}$$

$$\left[{FADH}^{-}\right]=\frac{a_{4}}{a_{1}+a_{2}+a_{3}+a_{4}}$$

where ${ABS}_{\exp}$ represents a measured absorption spectrum and ε is the molar absorption spectrum of one of the four states in Figure 1. $\left[{FAD}_{ox}\right]$ is the proportion of the FAD_ox_ state within the system. The range of fitting was 320 to 700 nm, except for *Cra*CRY in NADH, where the range was 390 to 700 nm. The fitting method was to search the coefficients a_1_, a_2_, a_3_ and a_4_ to minimize the discrepancy between the left-hand side and right-hand side of Equation 1. Two examples are provided in Fig. S2. The rates of photoreduction and re-oxidation were then fitted with Equation 2 below.

$${\left[{FAD}_{ox}\right]}_{t}=A\cdot e^{-\frac{t}{\tau }}+B$$

where ${\left[{FAD}_{ox}\right]}_{t}$ is the proportion of the FAD_ox_ state within the system at time t, τ is the lifetime of the FAD_ox_ state, and the rate of photoreduction is 1/τ. The fitting of the spectra was conducted using Matlab R2022a, and the kinetics were analyzed and fitted using Origin 2021.

Photoreduction extent was defined as the maximum difference in the proportion of the FAD_ox_ state before and after blue light illumination. Photoreduction efficiency is composed of the photoreduction rate and extent.

### Steady-state UV-Vis absorption and chemical reduction assays

For dithionite (DT) reduction, measurement of the spectra in the dark was started immediately after DT was added and mixed into the cuvette. The concentrated DT solution must be freshly prepared in a buffer containing HEPES (pH 7.4) and 150 mM NaCl. For 10 mM dithionite (DT) reduction, 10 μl of 200 mM DT was added to 190 μl of *Cra*CRY in the cuvette.

### Trypsin digestion assays

Trypsin (Gibco, 1.05 mM) was purchased from Thermo Fisher Scientific. Limited proteolysis of *Cra*CRY was performed as follows: to 90 μl of 0.6 mg/mL *Cra*CRY in a buffer containing HEPES (pH 7.4) and 150 mM NaCl, 10 μl of 8.75 μM trypsin was added and the mixture was incubated at room temperature in the dark. The digestion was subsequently quenched with 100 μl of loading buffer containing 400 μM trypsin inhibitor (Sigma–Aldrich) and 2% SDS. After 5 min of heating at 95 °C, 10 μl of the above mixture was loaded onto an SDS-PAGE gel. After conducting a preliminary experiment with various trypsin concentrations and digestion times, the chosen digestion conditions were 0.875 μM trypsin for 30 min.

For the lit samples, *Cra*CRY was exposed to a blue light LED 1 cm above the 1.5 ml tube to avoid heating the sample before the addition of trypsin. In a set of experiments, the aliquoted samples were exposed to blue light sequentially, with those first exposed to light being preserved in ice baths. All the samples were subsequently mixed with trypsin.

For the reducing-agent-treated *Cra*CRY, 12 mg/mL *Cra*CRY in a buffer containing a specific reducing agent was exposed to blue light for 3 min and then diluted to 0.6 mg/mL to avoid the effect of reducing agents on trypsin. The concentrations of TCEP, DTT, GSH, NADH, and NADPH used were 4 mM, 50 mM, 20 mM, 1 mM, and 1 mM, respectively. A control experiment was designed in which 12 mg/mL *Cra*CRY was exposed to blue light for 3 min and then diluted to 0.6 mg/mL with a buffer containing 2.5 mM DTT.

For samples treated with DT, 6 mg/mL *Cra*CRY was incubated for 12 min in buffer containing DT in the dark and then diluted to 0.6 mg/mL, followed by the addition of 8.75 μM trypsin at a ratio of 9:1 (v/v).

Gels were stained with Coomassie brilliant blue and imaged with Gel Imaging System (Gel Doc EZ, Bio-Rad), and various bands were quantified using Image Lab (version 4.0.1, Bio-Rad). Data from at least three independent experiments are plotted, and the SD of the mean is shown.

### Mass spectrometry of trypsin proteolysis bands

LC-MS was performed according to previous methods (17, 47). Protein gel pieces of wild-type *Cra*CRY in the light or the dark were analyzed using LC-MS (nanoEASY 1000, Thermo Fisher) and the column was an Acclaim PepMap RSLC (0.075 × 250 mm, Thermo Fisher). Raw MS data files were analyzed using Proteome Discoverer v1.4 (Thermo Fisher), and peptide identification was limited to *Cra*CRY.

### Size exclusion chromatography

For time-lapse dimerization under blue light, all samples were prepared at a concentration of 6 mg/mL in buffer containing HEPES (pH 7.4) and 150 mM NaCl. For concentration assays, all samples were prepared in buffer containing HEPES (pH 7.4) and 150 mM NaCl and the blue light illumination time was 4 min. The blue light illumination conditions were the same as those described for the absorption measurements. The Superdex 200 increase 10/300 column used for the SEC analysis was equilibrated with buffer containing HEPES (pH 7.4) and 150 mM NaCl. The experiments were performed at room temperature in darkness. The data were analyzed with AKTA software package (GE Healthcare), and the elution profiles monitored at 280 nm were normalized to the monomer peak.

### *In vitro* photorepair assays

The *in vitro* repair assay of *Cra*CRY was performed following published procedures (12, 53). The reaction mixture contained 40 μM irradiated (dT)18, 10 μM *Cra*CRY, 30 mM HEPES (pH 7.4), 4 mM Tris, and 90 mM NaCl. Additionally, in some experiments, 15 mM DTT was included as a control variable. A total of 200 μl of the mixture was placed in the cuvette, and the blue light LED at 100 W/m^2^ with an emission maximum at 450 nm was placed 1 cm above the cuvette. An Agilent 8453 spectrophotometer was used for recording the UV-Vis absorption spectra.

### Pull-down assays

His-tag-fused *Cra*CRY and GST-fused ROC15 (GARP) proteins were purified using His and GST affinity columns, respectively. Subsequently, 500 μl of 8 mg/mL *Cra*CRY and 8 mg/mL ROC15(GARP) mixture in the tube was exposed to blue light for 4 min. The sample was diluted with 10 ml of 50 mM HEPES buffer containing 150 mM NaCl and loaded into the GST affinity column. The samples of the mixture before loading (L), the flow-through solution (F), and the elution bound to the GST affinity column (E) were subsequently subjected SDS-PAGE. The band below GST-GARP was cleaved GST. Gels were stained with Coomassie brilliant blue and imaged with Gel Imaging System (Gel Doc EZ, Bio-Rad), and *Cra*CRY and GST-GARP bands were quantified using Image Lab (version 4.0.1, Bio-Rad). The interaction was quantitatively assessed based on the ratios of CraCRY to GST–GARP band intensities.

## Data availability

All data are contained in this article.

## Supporting information

This article contains supporting information.

## Conflict of interest

The authors declare that they have no conflicts of interest with the contents of this article.
