# Supplementary material for: An extra residue in the electron transfer chain holds the key for the dual functions of an animal-like cryptochrome
Source: J Biol Chem. 2025 Nov 13;302(1):110948. doi: 10.1016/j.jbc.2025.110948 (PMC12723161; doi:10.1016/j.jbc.2025.110948)
Supplement: Supporting Information [file mmc1.pdf]

Supporting Information

An extra residue in the electron transfer chain holds the key for the dual functions of an animal-like cryptochrome

Huaqiang Cheng[1+,] Xingbu Ding[1+], Yan-Wen Tan[1*]

Table of Contents

Supplementary Figures and Tables 2
Figure S1. Time-lapse absorption spectra of *Cra*CRY mutants. 2
Figure S2. The decomposition of absorption spectra. 3
Figure S3. Time-lapse absorption spectra of *Cra*CRY in the presence of reductants. 4
Figure S4. Photoreduction kinetics of WT in DTT. 5
Figure S5. Reoxidation spectral changes of *Cra*CRY-WT and mutants in the dark. 5
Figure S6. Reoxidation kinetics of *Cra*CRY-WT and mutants. 6
Figure S7. Reoxidation kinetics of *Cra*CRY-WT and Y373W in DTT. 7
Figure S8. Photoreduction and reoxidation lifetimes of *Cra*CRY. 7
Figure S9. Limited proteolysis of *Cra*CRY by trypsin. 8
Figure S10. Limited proteolysis of *Cra*CRY mutants by trypsin. 9
Figure S11. Limited proteolysis of wild type *Cra*CRY during reoxidation. 10
Figure S12. Limited proteolysis of H-bond mutants. 11
Figure S13. Chemical reduction kinetics of *Cra*CRY by DT. 12
Figure S14. Interactions of ROC15(GARP) with *Cra*CRY mutants. 13
Figure S15. Dimerization of *Cra*CRY changes with the increasing concentration. 14
Figure S16. Dimerization changes of *Cra*CRY with the increasing blue light exposure. 15
Figure S17. Kinetics of *Cra*CRY dimerization. 16
Figure S18. Dimerization of *Cra*CRY is stable during reoxidation. 16
Table S1. Photoreduction rate of *Cra*CRY. 17
Table S2. Photoreduction extent of CraCRY. 18
Table S3. Statistic tests of photoreduction extent of CraCRY. 18

Supplementary Figures and Tables

Figure S1. Time-lapse absorption spectra of *Cra*CRY mutants.

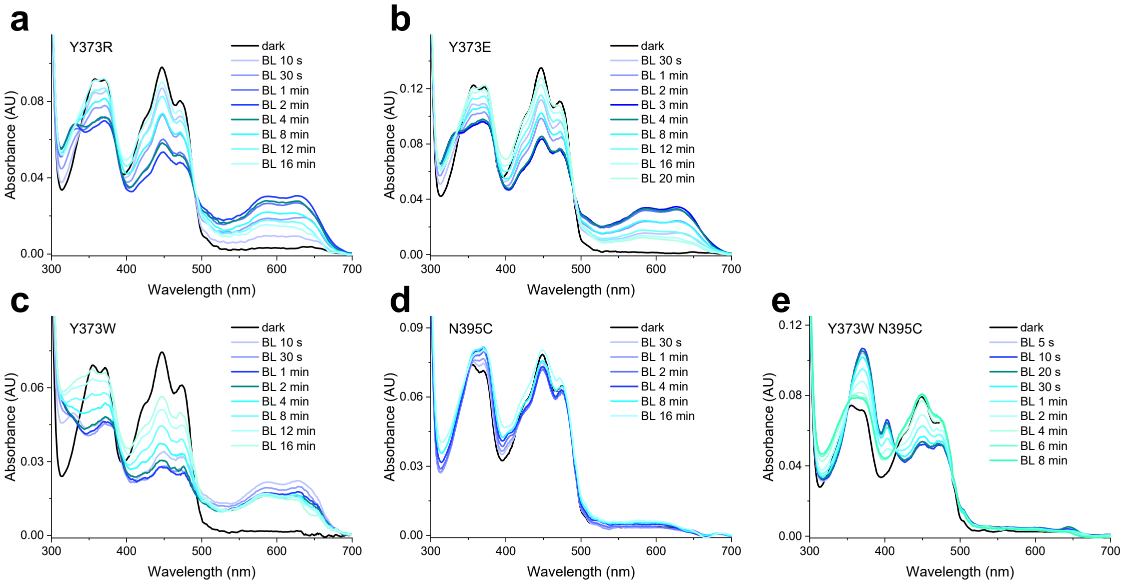

Figure S1. Time-lapse absorption spectra of *Cra*CRY mutants under blue light (BL) illumination in the absence of reducing agents.

Figure S2. The decomposition of absorption spectra.

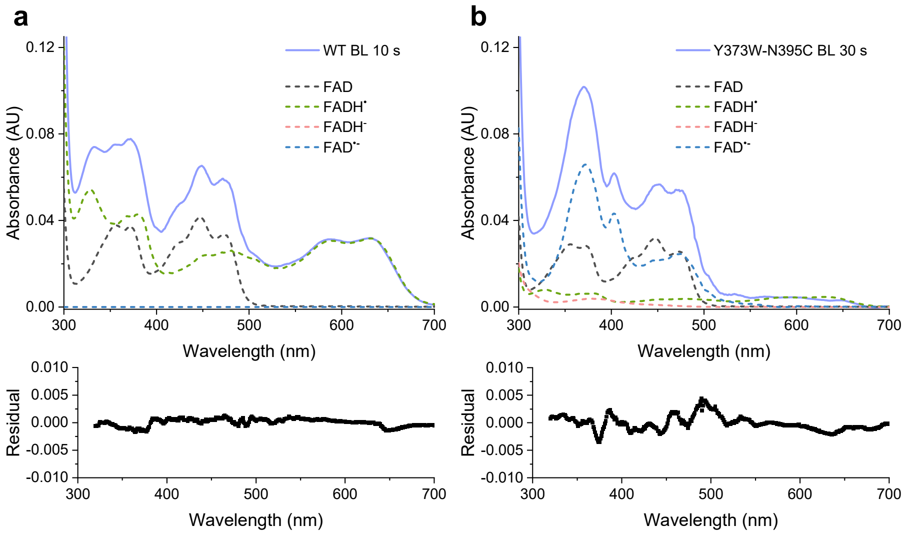

Figure S2. The decomposition of absorption spectra into four FAD states. (a) The decomposition of the absorption curve of *Cra*CRY-WT after 10 seconds of blue light illumination. The proportions of FAD, FADH•, FADH-, FAD•- are 0.36, 0.64, 0, 0, respectively. (b) The decomposition of the absorption curve of Y373W-N395C after 30 seconds of blue light illumination. The proportions of FAD, FADH•, FADH-, FAD•- are 0.35, 0.16, 0.05, 0.44, respectively. The lower panels display the residuals of the fitting results.

Figure S3. Time-lapse absorption spectra of *Cra*CRY in the presence of reductants.

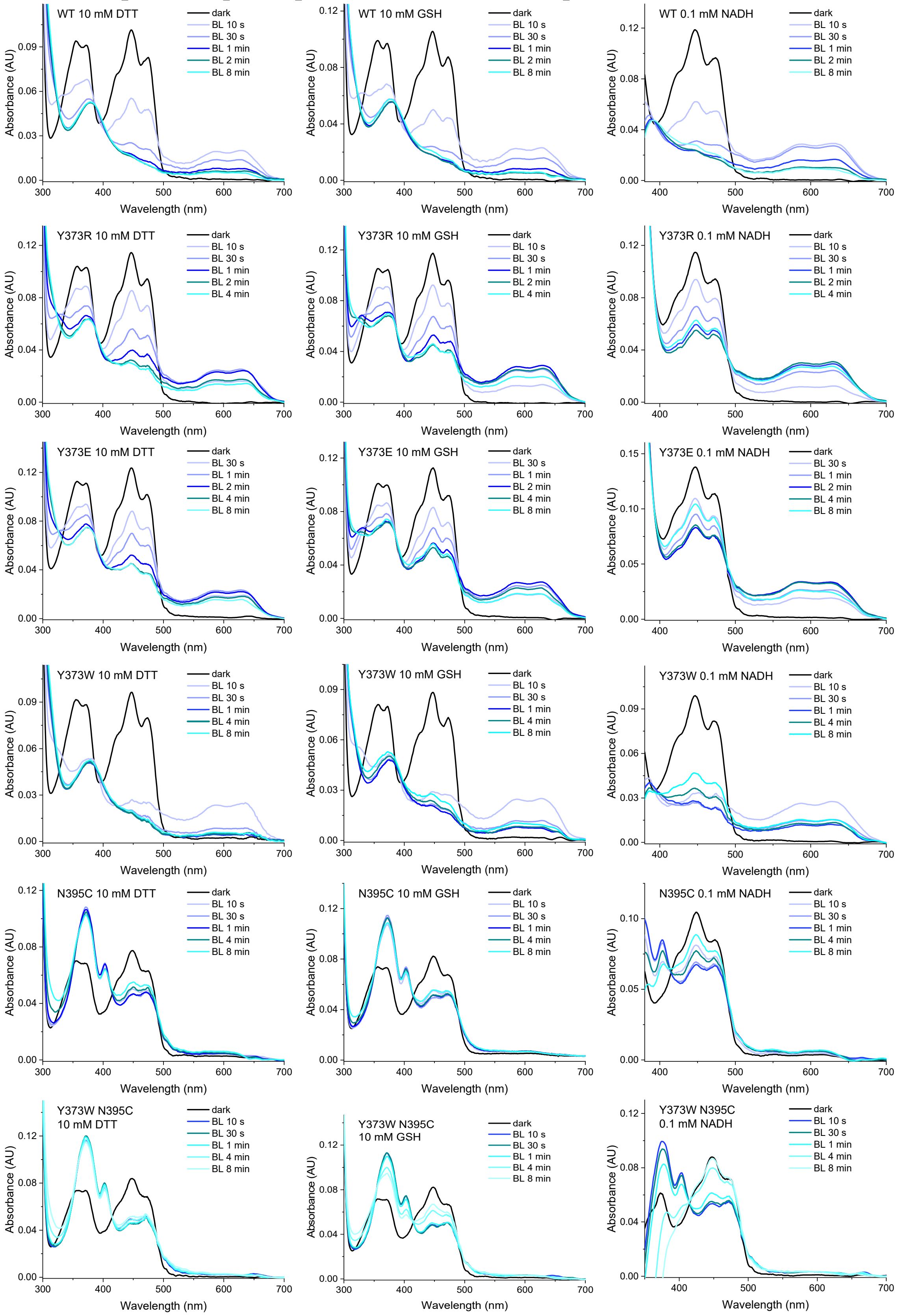

Figure S3. Time-lapse absorption spectra of *Cra*CRY-WT and mutants in the presence of reductants under blue light (BL). The range of the spectra in NADH is between 400 nm and 700 nm, because NADH exhibits absorbance below 400 nm.

Figure S4. Photoreduction kinetics of WT in DTT.

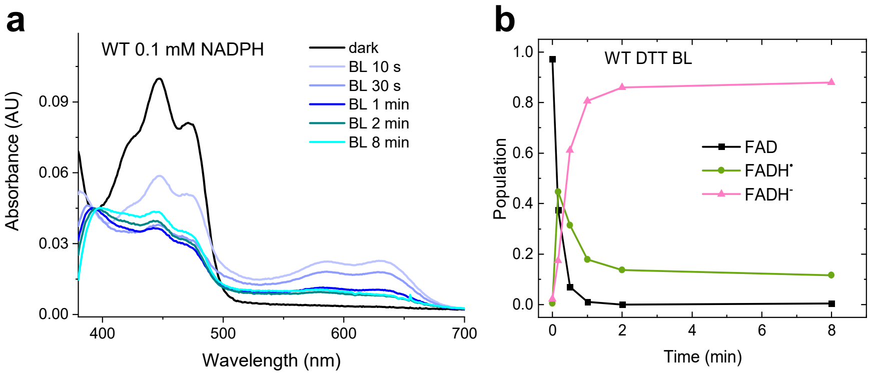

Figure S4. Time-lapse absorption spectra of WT in NADPH under blue light (a), and photoreduction kinetics of WT in DTT under 450 nm blue light (b). The spectral changes in NADPH are the same as those measured in NADH.

Figure S5. Reoxidation spectral changes of *Cra*CRY-WT and mutants in the dark.

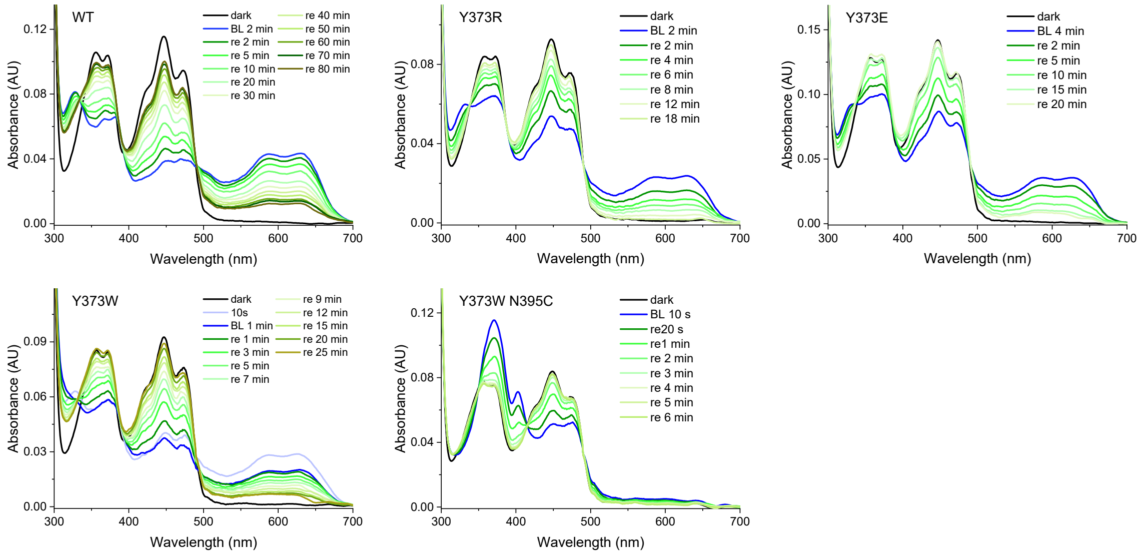

Figure S5. Reoxidation spectral changes of *Cra*CRY-WT and mutants in the dark. The samples were photoreduced under 450 nm blue light until saturation and then kept in the dark.

Figure S6. Reoxidation kinetics of *Cra*CRY-WT and mutants.

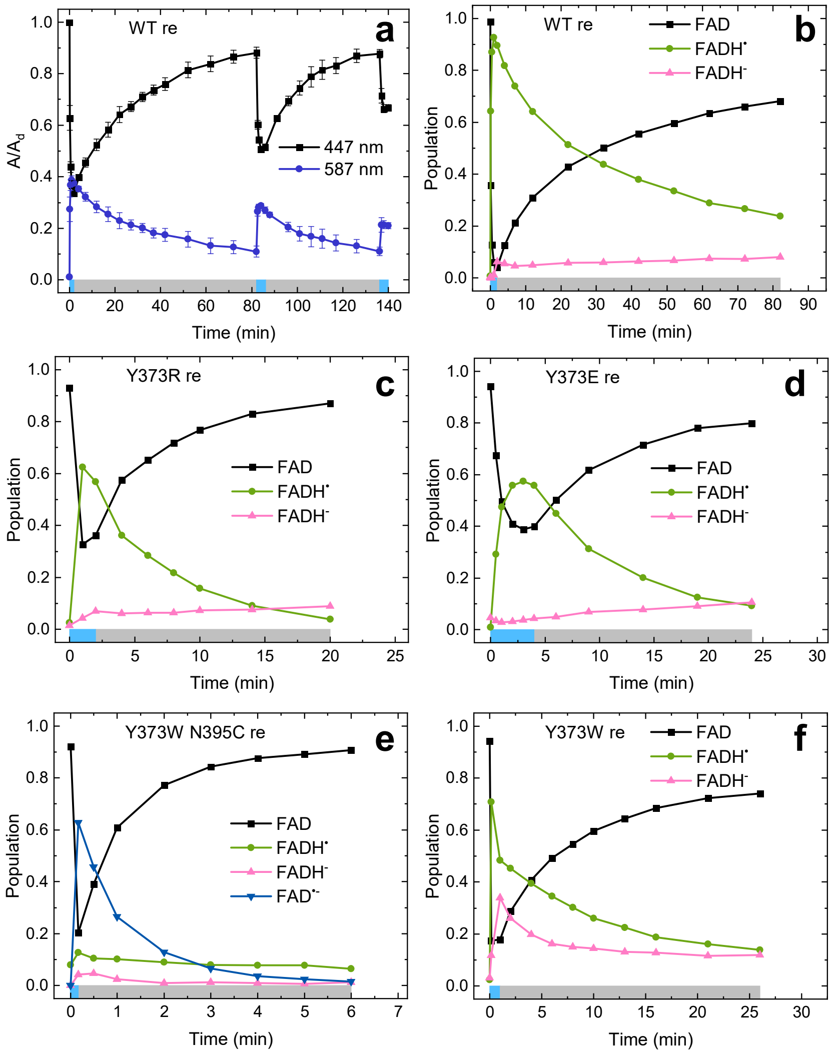

Figure S6. Reoxidation kinetics of *Cra*CRY-WT and mutants. (a) Kinetic traces at 447 nm and 587 nm for *Cra*CRY-WT illustrating the FAD_{ox} state and $FADH^•$ state. (b-f) Reoxidation kinetics of *Cra*CRY-WT and mutants. The samples were photoreduced under 450 nm blue light until saturation and then kept in the dark. The bars at the bottom of the figures indicate the samples are under blue light (blue) and in the dark (gray).

Figure S7. Reoxidation kinetics of *Cra*CRY-WT and Y373W in DTT.

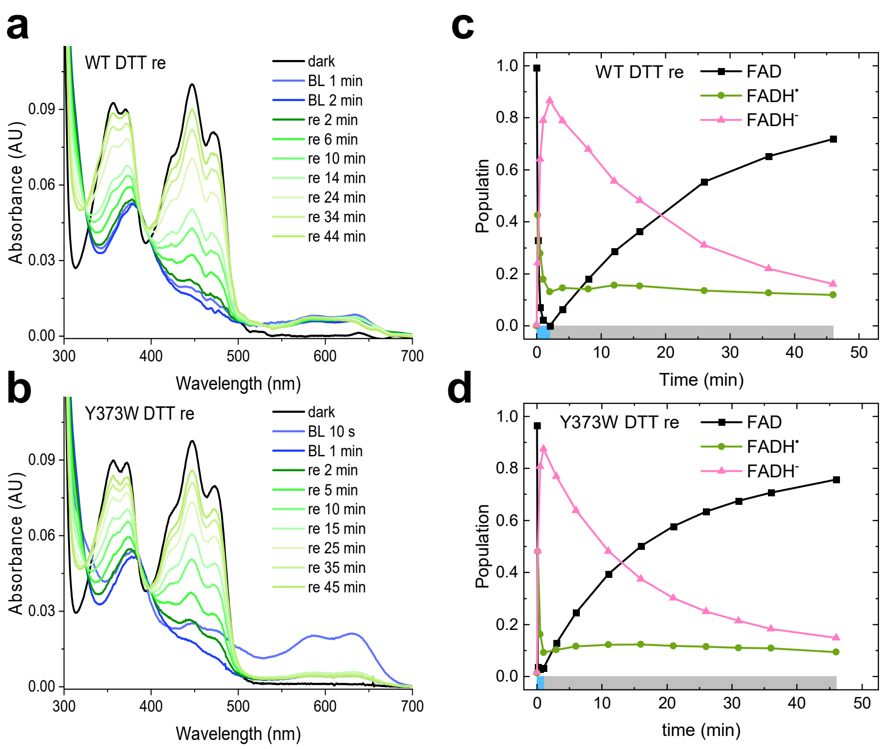

Figure S7. Reoxidation kinetics of *Cra*CRY-WT and Y373W in 10 mM DTT. Reoxidation spectral changes (a, b) and kinetics (c, d) of *Cra*CRY-WT and Y373W in the presence of DTT as a reducing agent. The samples were photoreduced with 450 nm blue light until saturation and then kept in the dark. The bars at the bottom of the figures indicate the samples are under blue light (blue) and in the dark (gray).

Figure S8. Photoreduction and reoxidation lifetimes of *Cra*CRY.

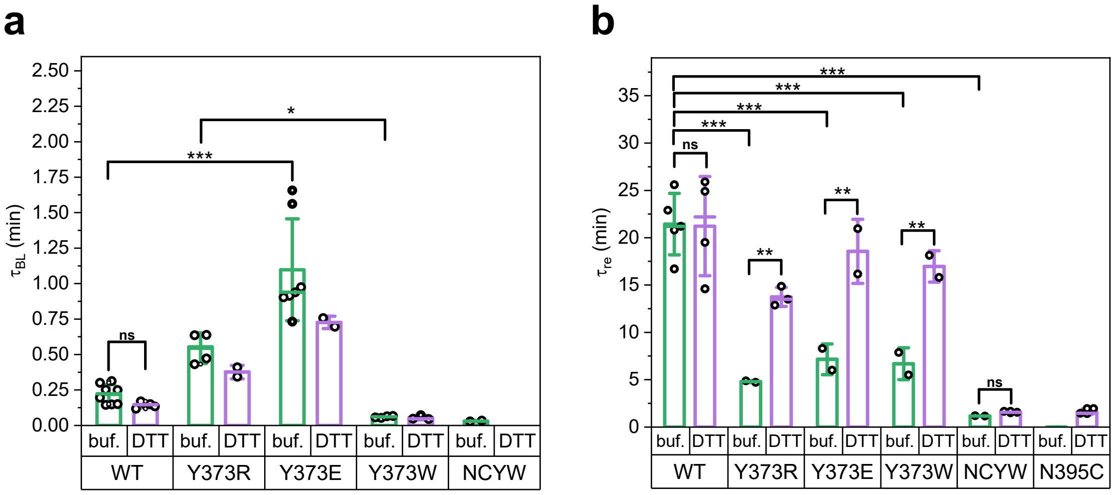

Figure S8. Photoreduction (a) and reoxidation (b) lifetimes of *Cra*CRY in the absence (buffer only) or presence of DTT as a reducing agent. The photoreduction of Y373W-N395C (NCYW) in DTT completed too rapidly to measure the lifetime. The photoreduction and reoxidation of N395C in the absence of DTT were not readily detectable. Data are the averages of at least two experiments, and error bars indicate the S.D. The data were analyzed by two-way ANOVA with Tukey's *post hoc* test; *p<0.05, **p<0.01, ***p<0.001; ns, not significant.

Figure S9. Limited proteolysis of *Cra*CRY by trypsin.

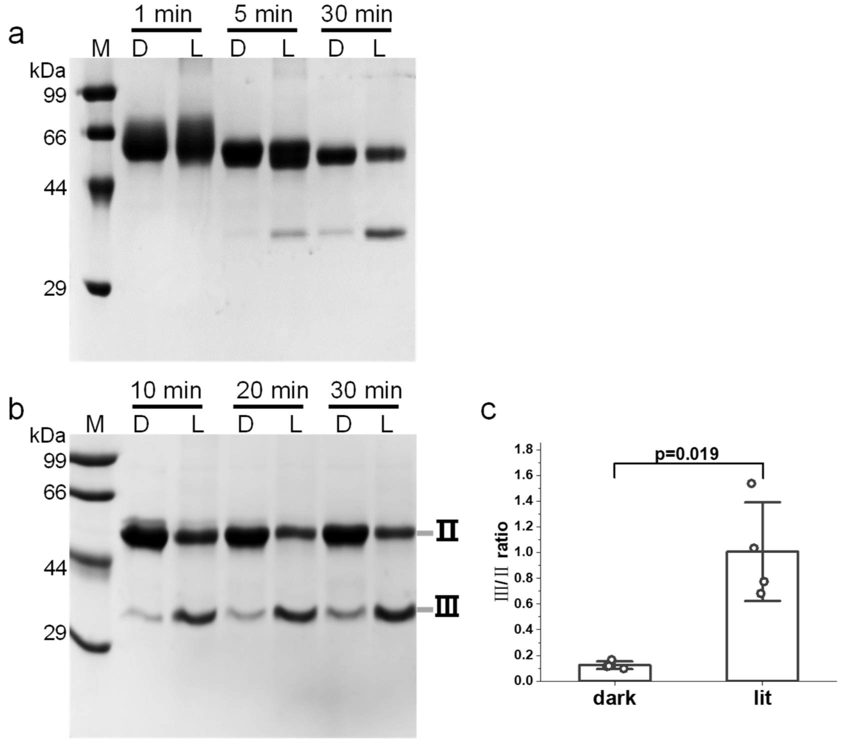

Figure S9. Limited proteolysis of *Cra*CRY by trypsin in the dark (D) and in the light (L). (a) Proteolysis of *Cra*CRY-WT with 0.44 μM trypsin for different incubation time. (b) Proteolysis of *Cra*CRY-WT with 0.88 μM trypsin for different incubation time. (c) Quantitative analysis of conformational changes using the ratio of band III to II with 0.88 μM trypsin for 30 min, which was used in the other proteolysis assays. The illumination time was 4 min. Data are the averages of 3 experiments, and error bars indicate the S.D. P values are shown for significant differences; two-tailed, unpaired t test.

Figure S10. Limited proteolysis of *Cra*CRY mutants by trypsin.

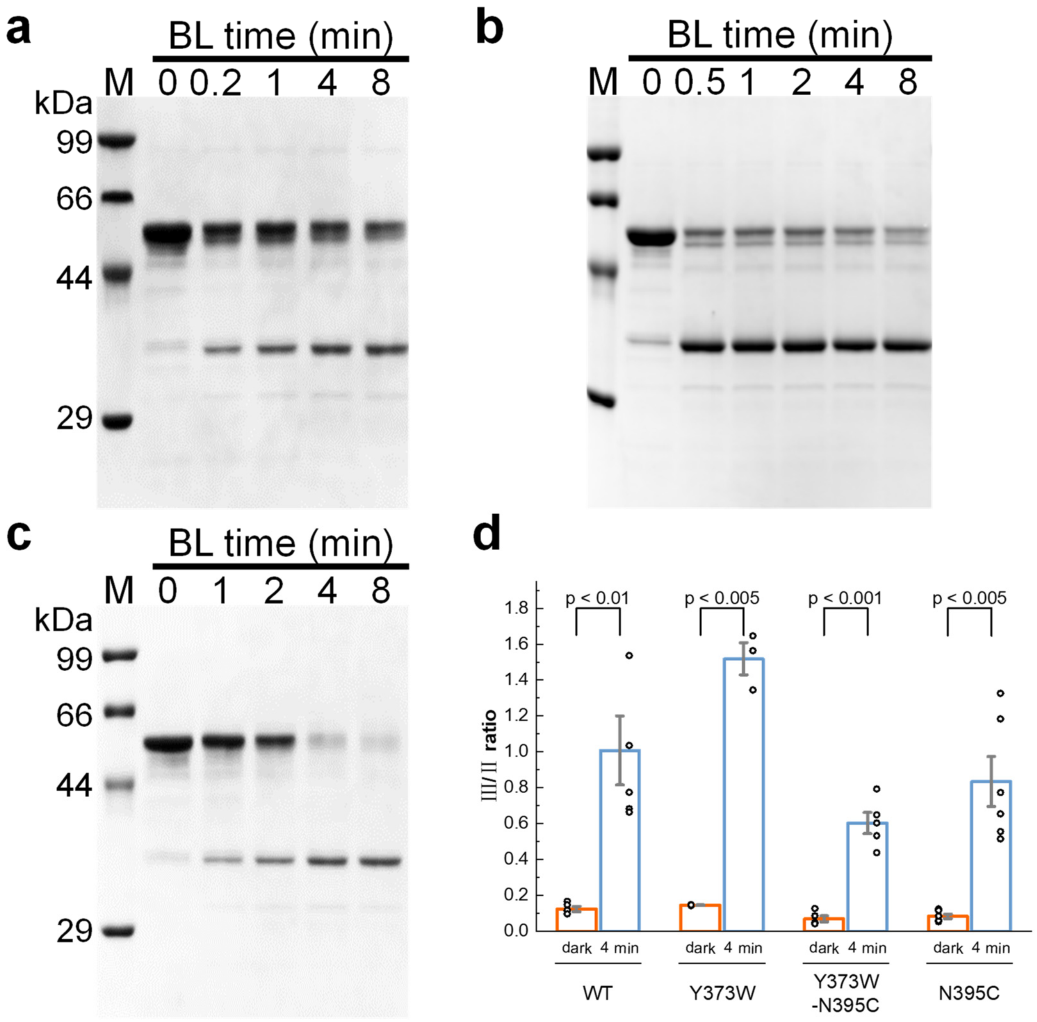

Figure S10. Limited proteolysis of *Cra*CRY mutants by trypsin. Y373W-N395C (a), Y373W (b), and N395C (c) were exposed to blue light for different time before adding trypsin and incubating in the dark for another 30 min. (d) The ratios of band III to band II in WT and three mutants were compared under dark conditions and after 4 minutes of blue light exposure. Error bars reflect the S.D. for $n \geqslant 3$. P values are shown for significant differences; two-tailed, unpaired t test.

Figure S11. Limited proteolysis of wild type *Cra*CRY during reoxidation.

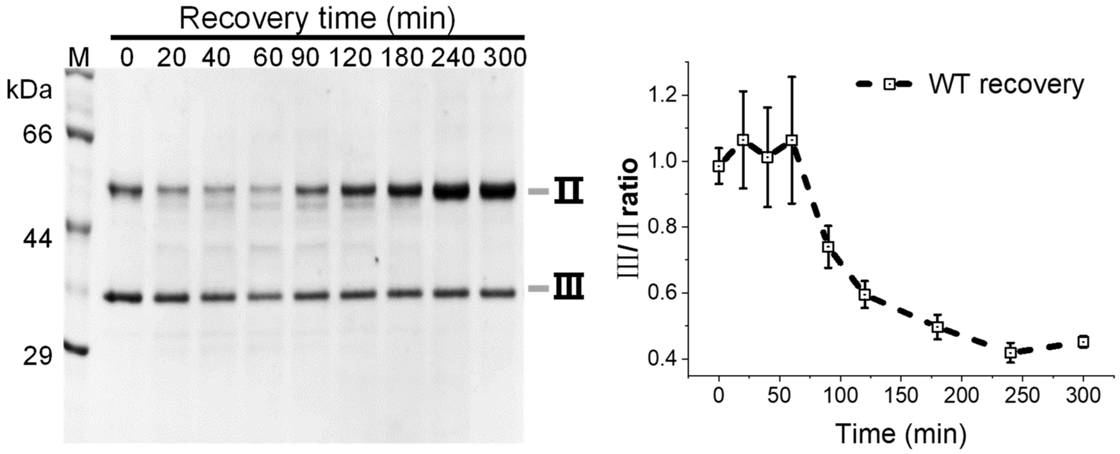

Figure S11. Limited proteolysis of wild type *Cra*CRY during reoxidation by trypsin and recovery of conformational changes. The samples were photoreduced with 450 nm for 4 min and then kept in the dark for different time. Data are the averages of 3 experiments, and bars indicate the S.D.

Figure S12. Limited proteolysis of H-bond mutants.

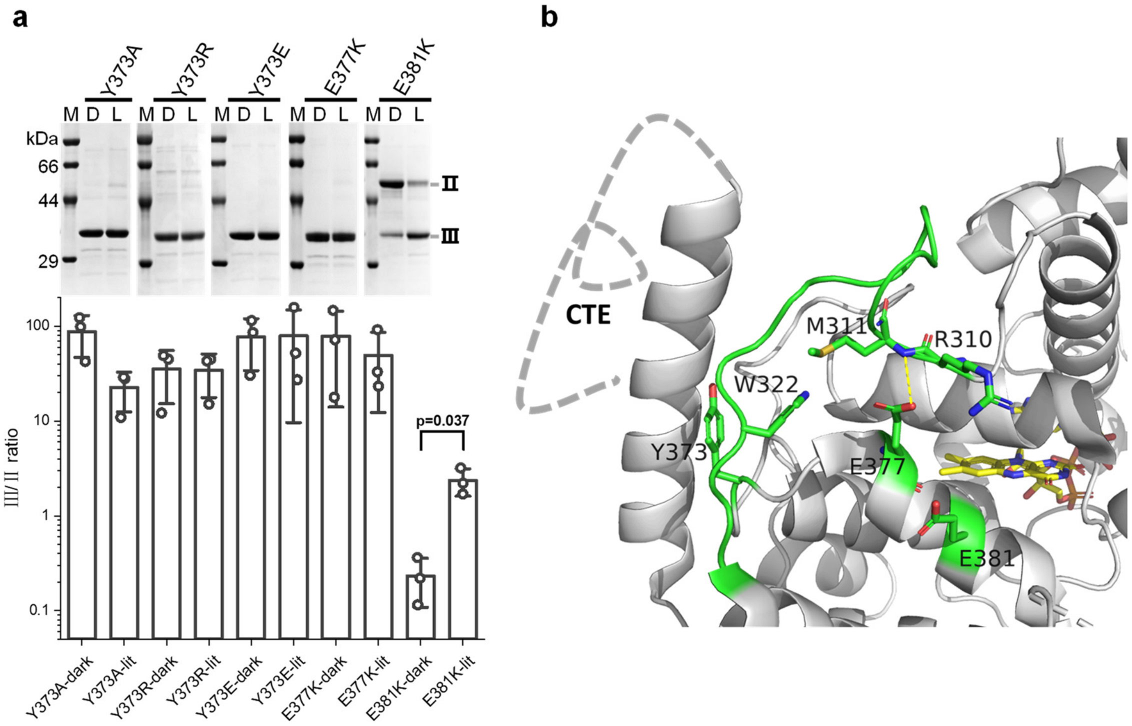

Figure S12. Limited proteolysis of *Cra*CRY mutants in the dark (D) and in the light (L). (a) The Y373 mutants and E377K under blue light and in the dark showing the similar proteolysis bands. The illumination time was 4 min. (b) The mutation sites are in the vicinity of Y373 and R310. The yellow dash line between E377 and R310 indicates the hydrogen bond (5zm0). And E381K is the control. M is marker. Error bars reflect the S.D.(n=3) P values are shown for significant differences; two-tailed, unpaired t test.

Figure S13. Chemical reduction kinetics of *Cra*CRY by DT.

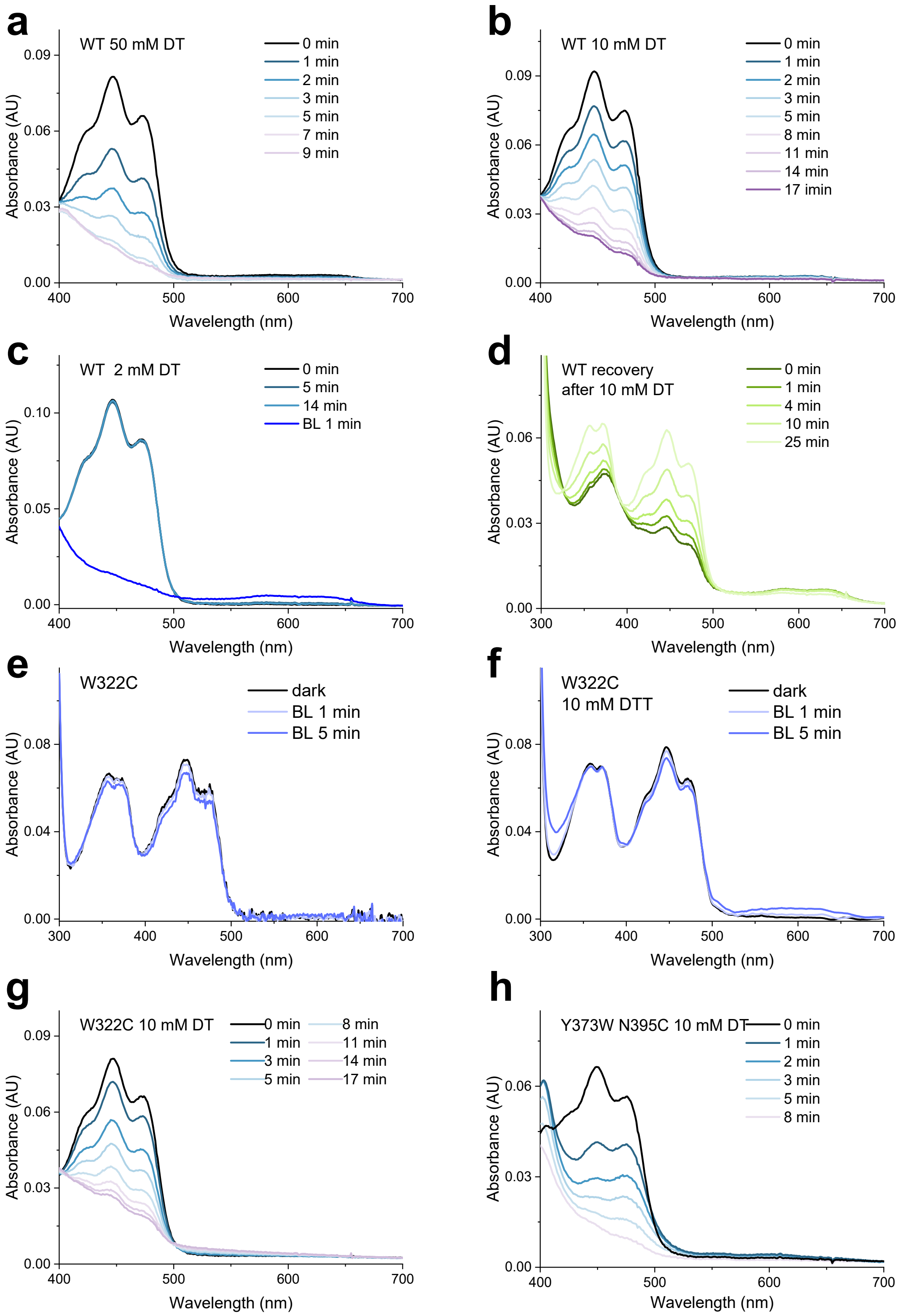

Figure S13. Chemical reduction kinetics of *Cra*CRY by DT (dithionite). (a, b) Wild type *Cra*CRY was reduced with 50 mM and 10 mM DT to the FADH⁻ state in the dark. (c) Wild type *Cra*CRY cannot be reduced with 2 mM DT unless under blue light. (d) Reduced *Cra*CRY by 10 mM DT was diluted in the normal buffer and recovered to the oxidation state. (e, f) Photoreduction of W322C in the absence or presence of DTT. (g, h) W322C and Y373W-N395C mutants were reduced to the FADH⁻ state with 10 mM DT in the dark.

Figure S14. Interactions of ROC15(GARP) with *Cra*CRY mutants.

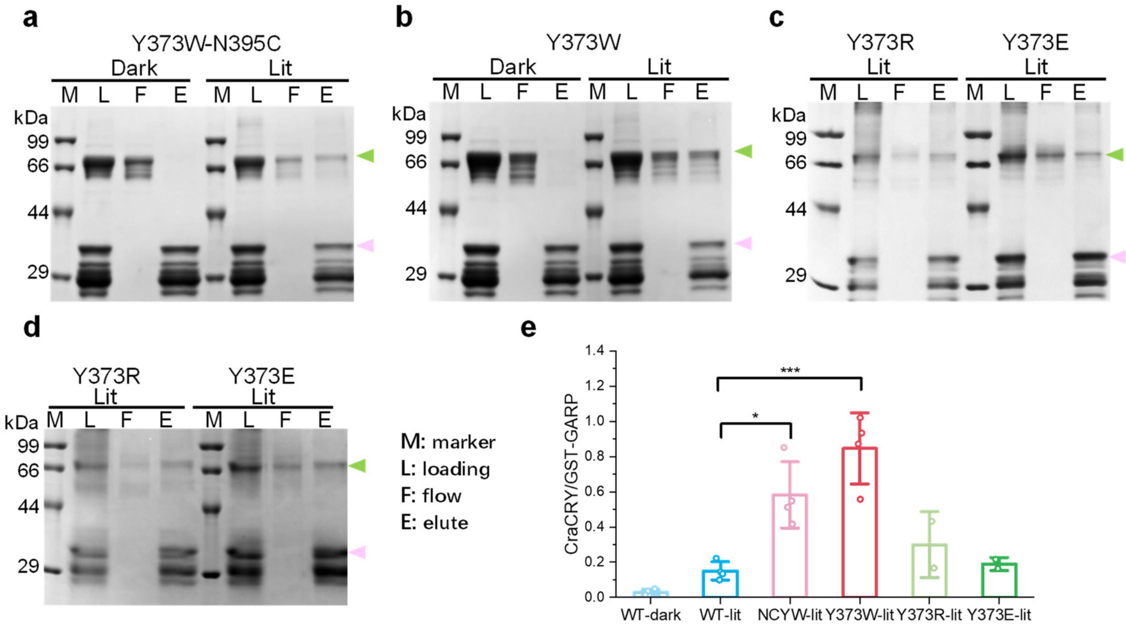

Figure S14. Interactions of ROC15(GARP) with *Cra*CRY mutants. Pull-down assay using SDS-PAGE for *Cra*CRY mutants under blue light was in the absence of reducing agents. The green arrows mark the band of *Cra*CRY and mutants. The pink arrows mark the band of GST-tagged ROC15(GARP). The blue light exposure time was 4 minutes. The results of the pull-down assays for *Cra*CRY depend on the protein states. *Cra*CRYs are prone to degradation, and the GST fusion linkage is fragile. The loading and elution lanes after illumination showed diffuse bands with molecular weights higher than *Cra*CRY. (a, b) Pull-down assays of Y373W-N395C and Y373W in the dark and under blue light. (c, d) Pull-down assays of Y373R and Y373E under blue light. (e) Error bars reflect the S.D. from at least two independent experiments. The data were analyzed by one-way ANOVA with Tukey's *post hoc* test; *$p<0.05$, **$p<0.01$, ***$p<0.001$.

Figure S15. Dimerization of *Cra*CRY changes with the increasing concentration.

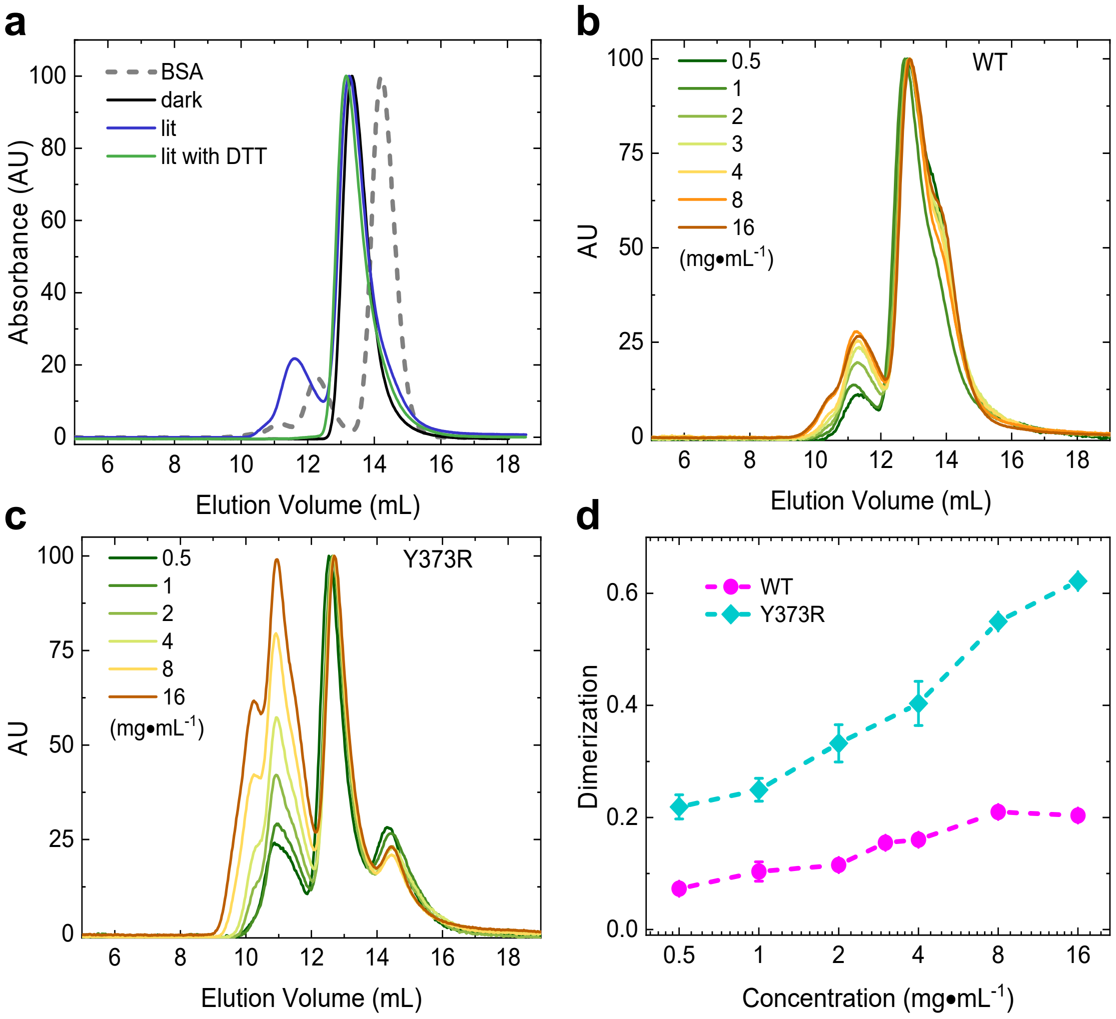

Figure S15. Dimerization of *Cra*CRY changes with the increasing concentration shown by SEC. WT and Y373R were illuminated for 4 min by blue light. Dimerization is inhibited by DTT. The shoulders at the elution volume of 14-16 mL corresponded to a small amount of degraded protein. Integration area of dimer elution peaks is used to defined the oligomeric fraction within the whole *Cra*CRY elution area. Data are the averages of 2-3 experiments, and error bars indicate S.D.

Figure S16. Dimerization changes of *Cra*CRY with the increasing blue light exposure.

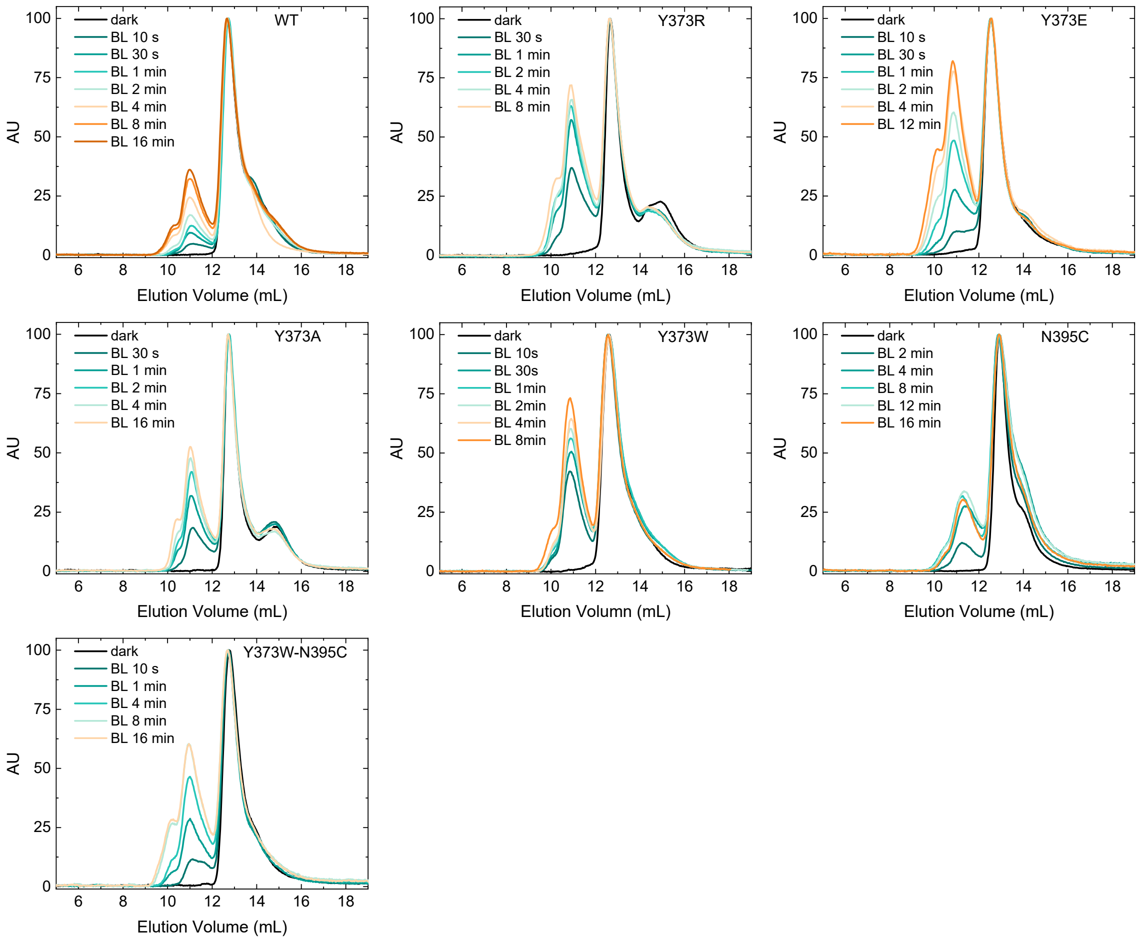

Figure S16. Dimerization changes of *Cra*CRY-WT and mutants with the increasing blue light (BL) exposure. The shoulders in SEC profiles at the elution volume of 14-16 mL corresponded to a small amount of degraded protein.

Figure S17. Kinetics of *Cra*CRY dimerization.

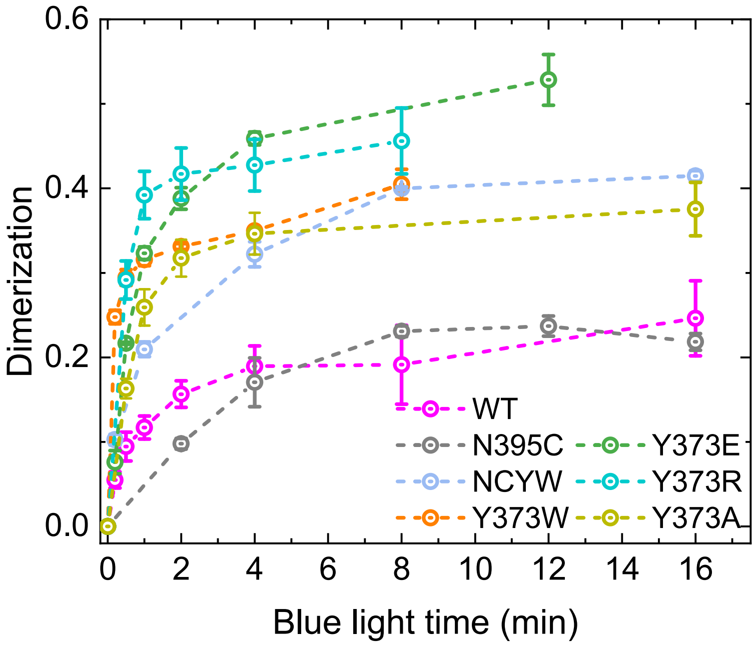

Figure S17. Kinetics of *Cra*CRY dimerization. Integration area of dimer elution peaks in SEC is used to defined the oligomeric fraction within the whole *Cra*CRY elution area. Data are the averages of 2-3 experiments, and error bars indicate S.D.

Figure S18. Dimerization of *Cra*CRY is stable during reoxidation.

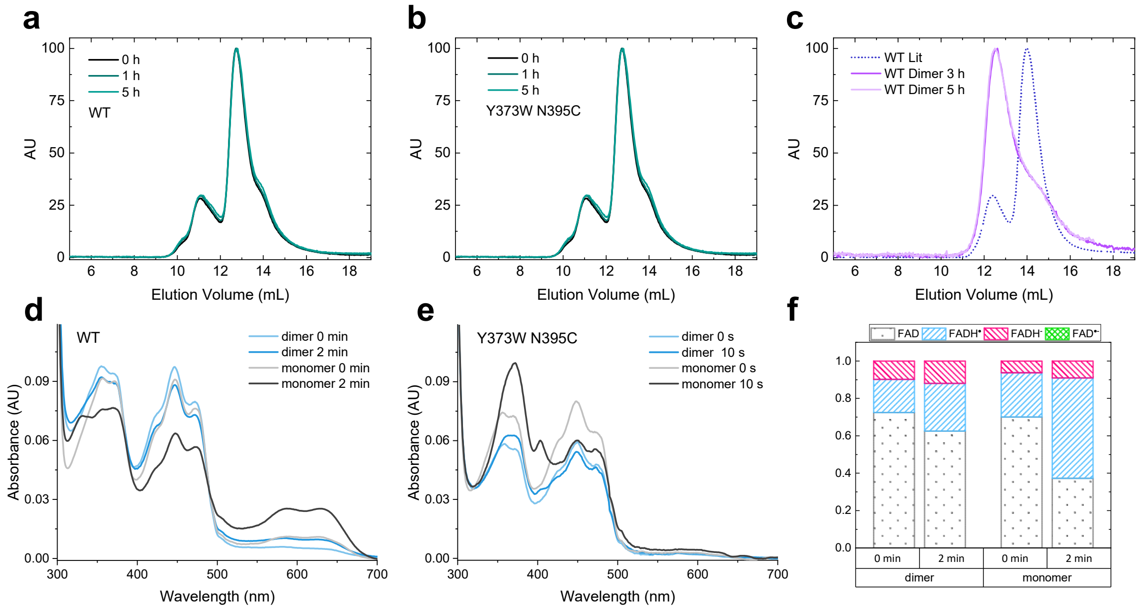

Figure S18. Dimerization of *Cra*CRY is stable during reoxidation. Photoreduced *Cra*CRY-WT (a) and Y373W-N395C (b) were placed in the dark for 0 h, 1h, and 5 h and then analyzed by SEC. (c) *Cra*CRY dimer collected by SEC was analyzed by second round SEC after 3 h and 5 h in the dark. (d-f) Dimer and monomer were collected seperately after SEC, and illuminated with 2 min blue light for the second time. Absorption spectra were detected before and after illumination for WT (d) and Y373W-N395C (e). (f) Spectra of WT in d were decomposed into FAD states. The time for the first illumination is 4 min.

Table S1. Photoreduction rate of *Cra*CRY.

The lifetimes (min) of photoreduction and reoxidation are determined based on changes in the FAD_{ox} state. The photoreduction of Y373W and Y373W-N395C (NCYW) in the presence of DTT occurs too rapidly to measure their lifetimes.

samples	τ_{BL}	S.D.	τ_{re}	S.D.
WT	0.22	0.06	21.4	2.9
Y373R	0.54	0.09	4.8	0.1
Y373E	1.10	0.26	7.2	1.2
Y373W	0.06	0.01	6.7	1.2
Y373W-N395C	0.03	0.00	1.2	0.0
N395C	1.23	0.18		
WT DTT	0.14	0.02	21.2	4.5
Y373R DTT	0.38	0.03	13.7	0.8
Y373E DTT	0.73	0.03	18.6	2.4
Y373W DTT	0.04	0.01	17.0	1.2
Y373W-N395C DTT			1.57	0.07
N395C DTT			1.61	0.24

Table S2. Photoreduction extent of *Cra*CRY.

The extent of photoreduction is expressed as 1 – [FAD_{ox}], where [FAD_{ox}] is the minimum proportion of the oxidized FAD state observed during photoreduction (Figure 3), measured either in the normal buffer or in the presence of reductants (10 mM DTT, 10 mM GSH, or 0.1 mM NADH). Error bars reflect the S.D. from at least three independent experiments.

Samples	Buffer	DTT	GSH	NADH
WT	95.4%±5.6%	99.6%±0.4%	97.9%±1.3%	96.2%±1.6%
Y373R	69.8%±2.5%	88.7%±2.2%	78.3%±1.2%	74.2%±3.4%
Y373E	60.4%±2.8%	83.8%±2.8%	68.2%±1.8%	66.2%±1.1%
Y373W	85.4%±1.1%	94.6%±3.7%	91.8%±1.2%	87.9%±1.1%
Y373W-N395C	76.5%±3.4%	89.7%±1.9%	83.0%±0.7%	76.9%±1.8%
N395C	27.9%±2.5%	82.9%±1.2%	85.5%±2.4%	72.4%±1.5%

Table S3. Statistic tests of photoreduction extent of *Cra*CRY.

The table summarizes the p values for the samples shown in Figure 3 and discussed in the article. P values are shown for significant differences; two-tailed, unpaired t test. For example, the p value comparing the proportion of WT in the $FADH^-$ state with 10 mM DTT to that in the absence of reductants is 5.6E-09.

Samples	p value	Samples	p value
WT buffer $FADH^•$<br>Y373R buffer $FADH^•$	0.0034	WT buffer $FADH^-$<br>WT DTT $FADH^-$	5.6E-09
WT buffer $FADH^•$<br>Y373E buffer $FADH^•$	0.00069	Y373R buffer $FADH^-$<br>Y373R DTT $FADH^-$	4.9E-06
WT buffer $FADH^-$<br>Y373W buffer $FADH^-$	2.4E-08	Y373E buffer $FADH^-$<br>Y373E DTT $FADH^-$	9.8E-06
WT buffer $FAD^{•-}$<br>NCYW buffer $FAD^{•-}$	0.00011	Y373W buffer $FADH^-$<br>Y373W DTT $FADH^-$	2.1E-10
WT buffer $FAD^{•-}$<br>N395C buffer $FAD^{•-}$	0.0089	NCYW buffer $FAD^{•-}$<br>NCYW DTT $FAD^{•-}$	0.0012
N395C buffer $FAD^{•-}$<br>NCYW buffer $FAD^{•-}$	2.3E-05	N395C buffer $FAD^{•-}$<br>N395C DTT $FAD^{•-}$	3.7E-05
WT DTT $FADH^-$<br>Y373W DTT $FADH^-$	0.53	Y373E DTT $FADH^-$<br>Y373E GSH $FADH^-$	0.032
N395C DTT $FAD^{•-}$<br>NCYW DTT $FAD^{•-}$	0.076	Y373E GSH $FADH^-$<br>Y373E NADH $FADH^-$	0.19
